# Identification and Validation of N6-Methyladenosine-Related Biomarkers for Bladder Cancer: Implications for Immunotherapy

**DOI:** 10.3389/fonc.2022.820242

**Published:** 2022-03-02

**Authors:** Hongyu Deng, Faqing Tang, Ming Zhou, Dongyong Shan, Xingyu Chen, Ke Cao

**Affiliations:** ^1^Department of Clinical Laboratory, Hunan Cancer Hospital, The Affiliated Cancer Hospital of Xiangya School of Medicine, Central South University, Changsha, China; ^2^Cancer Research Institute and School of Basic Medical Sciences, Central South University, Changsha, China; ^3^NHC Key Laboratory of Carcinogenesis, Hunan Key Laboratory of Oncotarget Gene, Hunan Cancer Hospital, The Affiliated Cancer Hospital of Xiangya School of Medicine, Central South University, Changsha, China; ^4^Department of Oncology, Third Xiangya Hospital, Central South University, Changsha, China

**Keywords:** bladder cancer, N6-methyladenosine, tumor immune microenvironment, anti-PD-L1, immune checkpoint inhibitors

## Abstract

N6-methyladenosine (m^6^A) has emerged as one of the most important modifications of RNA. Based on the expression of 23 different modes of m^6^A regulatory factors, we identified three different m^6^A modification patterns in bladder cancer. The effects of the three different modes of m^6^A modification on clinicopathological characteristics, immune cell infiltration levels and expression levels of immune checkpoint genes were comprehensively analyzed. In addition, the effects of different modes of m^6^A modification on the therapeutic efficacy of anti-PD-L1 immunotherapy (atezolizumab) are also discussed. Our results confirm that m^6^A methylation plays an important role in immune cell recruitment in the tumor microenvironment of bladder cancer, which influences the efficacy of anti-PD-L1 therapy for bladder cancer. We further confirmed the important role of FTO protein in the biological function of bladder cancer cells by performing *in vitro* experiments. FTO functions as an oncogene in bladder cancer cells, and upon FTO knockdown, the level of m^6^A enzyme activity in bladder cancer cells was significantly increased, apoptosis was increased, and cell proliferation and cell invasion were reduced. In addition, our study also confirmed that K216H and K216E are probably important targets for regulating FTO. We provide new insights into the regulatory pathways of the immune microenvironment and the methylation function of m^6^A in bladder cancer, which will help in designing novel diagnostic methods, prognostic tools, and therapeutic targets.

## Introduction

Bladder cancer is the most common cancer of the urinary system and is characterized by a difficult early diagnosis, rapid metastasis, and resistance to treatment. Immune checkpoint therapy (ICT) targets immune pathway effectors such as cytotoxic lymphocyte antigen-4 (CTLA-4), programmed cell death protein 1 (PD-1), or programmed death-ligand 1 (PD-L1) (1). Anti PD-L1 immunotherapy has been approved by the Food and Drug Administration (FDA) for the treatment of metastatic bladder cancer (2, 3). Immunotherapeutics that target PD-1 or PD-L1 have significantly improved the survival outcomes for some patients, showing an astonishing effect in a small number of patients with sustained efficacy. The improved clinical efficacy for the detection of cancer of the urinary system including advanced-stage bladder cancer has changed the intervention measures used (4); however, some patients with bladder cancer undergoing immunotherapy show no response to ICT or display resistance to drugs, and this scenario does not meet the clinical needs of these patients (5, 6). At present, multiple studies have confirmed that the immune response resulting due to many tumors, including those found in bladder cancer, is related to the level of immune cell infiltration in the tumor microenvironment, the expression of PD-1/PD-L1, and the tumor mutation burden (TMB) (7, 8).

The microenvironment in which tumor cells grow and survive is called the tumor microenvironment (TME), which includes stromal cells such as cancer-related fibroblasts and macrophages, immune infiltrating cells (myeloid cells and lymphocytes), bone marrow-derived cells (BMDCs) such as endothelial progenitor cells and hematopoietic progenitor cells, and secretory factors such as cytokines (9). There is mounting evidence that TME plays a crucial role in tumor progression, immune escape, and has an impact on the response to immunotherapy (10, 11). A comprehensive analysis of the heterogeneity and complexity of the TME is a key step in improving the success rate of existing ICTs and the development of new immunotherapy strategies. Further exploration of the regulatory mechanisms underlying TME cell infiltration will improve the ability to guide and predict the response to immunotherapy, and may help identify new therapeutic targets.

N6-methyladenosine (m^6^A) is the most common and abundant RNA modification in eukaryotes. As a reversible epigenetic modification, m^6^A is found in almost all types of RNAs, including mRNAs, ribosomal RNAs (rRNAs), long noncoding RNAs (lncRNAs), microRNAs (miRNAs), small nuclear RNAs (snRNAs), and circular RNAs (circRNAs), and are dynamically regulated in many physiological processes (12, 13). The m^6^A modification is a dynamic and reversible process in mammalian cells and is regulated by various methyltransferases, demethylases, and binding proteins, also known as writers, erasers, and readers, respectively. The process of m^6^A methylation is catalyzed by methyltransferases such as RBM15, ZC3H13, METTL3, METTL14, WTAP, and KIAA1429, and the removal process is mediated by demethylases such as FTO and ALKBH5 (14). Further, a group of specific RNA binding proteins, such as YTHDF1/2/3, YTHDC1/2, HNRNPA2B1, and IGF2BP1/2/3 can recognize the m^6^A motif, thus affecting the function of m^6^A (15). Results from various studies have shown that the abnormal expression and changes to gene expression for m^6^A regulatory factors are related to the dysregulation of multiple biological processes essential for the initiation, progression, metastasis, drug resistance, recurrence, and immune regulation of cancer (16).

Many studies have confirmed the special correlation between m^6^A modification and changes in the TME. Li et al. (17) observed that when METTL3 was depleted, the steady-state growth of T cells was blocked and was maintained at a slow rate. Further, their research showed that METTL3 deletion resulted in a decrease in the level of m^6^A, and an increase in the mRNA stability and protein levels of SOCS1, SOCS3, and CISH, thus negatively regulating the IL-7 signal production in CD4^+^T cells. Results from another study (18) revealed the role of m^6^A in dendritic cell activation, in which METTL3 mediated the m^6^A modification of the transcripts for the adaptor Tirap for CD40, CD80, and TLR4. Signal adaptors enhanced the translation of METTL3 in dendritic cells, thereby enhancing TLR4/NF-κB signal transduction, producing cytokines, and stimulating T cell activation. From the perspective of immune checkpoint therapy, the m^6^A binding protein YTHDF1 can be used to modulate antitumor immunity and improve immunotherapy, as it can regulate the expression of lysosomal proteinase in an m^6^A dependent manner. YTHDF1 can recognize the transcription of m^6^A modified transcripts and encodes a lysosomal protease to increase translation in dendritic cells. Moreover, the deletion of *YTHDF1* enhances the cross-presentation of tumor antigens and the cross initiation of CD8^+^T cells *in vivo*. Further, Wang et al. confirmed that the deletion of *YTHDF1* enhances the therapeutic effect of the PD-L1 checkpoint blockade (19).In melanoma, elevated *FTO* expression levels promote tumor growth by reducing the methylatin of m^6^A in the mRNA for PD-1 (*PDCD1*), *CXCR4*, and *SOX10* and preventing their RNA decay mediated by YTHDF2. Knockout of *FTO* in melanoma cells induces tumor cells to become sensitive to interferon-gamma (IFNγ) *in vitro* and promotes a sensitizing response of melanoma cells to anti-PD-1 antibody in mice (20). Together, these data prove that the m^6^A modification plays an important role in regulating the tumor immune microenvironment, and the combined use of m^6^A modulators and PD-1/PD-L1 drugs may prove to be a new strategy to enhance the clinical efficacy of tumor immunotherapy.

In this study, we examined the effect of the m^6^A methylation modification on the invasion characteristics of immune microenvironment cells and the effect of anti-PD-L1 therapy on bladder cancer. Our study helps elucidate the molecular mechanism underlying the regulation of the immune microenvironment in bladder cancer and provides new predictors, potential auxiliary targets, and directions for more effective immunotherapy strategies for combating bladder cancer.

## Materials and Methods

### Datasets

The gene expression profile was measured experimentally using the Illumina HiSeq 2000 RNA Sequencing platform at the University of North Carolina The Cancer Genome Atlas (TCGA) genome characterization center. Level 3 data were downloaded from the TCGA data coordination center. This dataset shows the gene-level transcription estimates, i.e., the log_2_ (x+1) transformed RSEM normalized counts, and somatic mutation data was downloaded from the site https://portal.gdc.cancer.gov/. IMvigor 210 cohort is a multicenter, single-arm phase II clinical study for evaluating the safety and efficacy of Tecentriq, a PD-L1 inhibitor, in patients with advanced urothelial carcinoma (21, 22). The complete processed expression data, detailed clinical annotations, and somatic mutation data were obtained from IMvigor210CoreBiologies, which is a complete documentation software and data package for the R statistical computing environment. The software package is available free of charge under the Creative Commons 3.0 License.

### Consensus Clustering for 23 m^6^A Regulators

We identified 23 m^6^A regulators from published literature (23, 24), including 2 erasers (*ALKBH5*, *FTO*), 13 readers (*FMR1*, *HNRNPA2B1*, *HNRNPC*, *IGF2BP1*, *IGF2BP2*, *IGF2BP3*, *YTHDC1*, *YTHDC2*, *YTHDF1*, *YTHDF2*, *YTHDF3*, *ELAVL1*, *LRPPRC*), and 8 writers (*KIAA1429*, *METTL14*, *METTL3*, *RBM15*, *RBM15B*, *WTAP*, *ZC3H13*, *CBLL1*). The consensus clustering algorithm determined the optimal number of clusters (K value) as 3. Based on the expression of the 23 m^6^A modulators, different m^6^A modification patterns were identified by using k-means (based on Euclidean distance), and the patients were classified into three groups for further analysis. We used the consusclusterplus R package to perform the above steps, and performed 1000 iterations (50 iterations, resampling rate of 80%) to ensure the stability of the classification (25).

### Common Molecular Typing of Bladder Cancer

We used several published subtype classification systems for bladder cancer, including the Baylor subtype described by Mo et al. (26), who defined urothelial differentiation based on 18 gene markers, thus dividing the muscle-invasive bladder cancers (MIBC) and non-MIBCs (NMIBC) into basal and differentiated subgroups. Damrauer et al. (27) identified basal and luminal cancer subtypes by consensus cluster analysis and further identified 47 genes as predictors of these subtypes. The MDA subtype (28) was analyzed by using 2252 genes (2697 probes) from 73 freshly frozen primary MIBC samples, including p53 like, luminal, and basal cancer. The Lund group (29) presented a six-class system based on global mRNA expression, including the urothelial-like, genomically unstable, epithelial infiltrated, SCC-like/Mes-like, SCC-like/UroB, and Sc/NE-like cancer. TCGA genotyping (30) used 2707 genes to conduct unsupervised cluster analysis on 129 patients with MIBC. The patients were classified into four subtypes (I, II, III, and IV), using the Cartes d’Identitédes Tumeurs (CIT)-Curie subtypes (31). These classifiers were combined into an R package (BLCA subtyping) (32), which can be found at https://github.com/cit-BioInfo/BLCAsubtype. We applied these classifiers independently to the TCGA-BLCA cohort and the IMvigor210 cohort to analyze the relationship between the subtypes of different classification methods and three different m^6^A modification patterns. Further, we interpreted the differences among the three different m^6^A modified subtypes.

### Estimation of TME Cell Infiltration

We used the single-sample gene set enrichment analysis (ssGSEA) algorithm to quantify the relative abundance of cell infiltration in TME. We obtained gene sets from the data of Qingzhu Jia et al. (33) to label the different types of TME infiltrating immune cell types. As demonstrated by Jia et al. (33), we calculated the antitumor-immunity-score as the sum of the immune cell scores of various antitumor cells. Further, the pro-tumor-immunity-score is the sum of the scores of tumor-promoting immune cells, and we define the tumor immunity score as the difference between the pro-tumor-immunity score and the antitumor-immunity-score for unified analysis. In addition, we also used the ESTIMATE algorithm (34) to estimate the ratio of the immune matrix components of each sample in the TME to further explore the differences of TME components including immune cells and stromal cells scores among different m^6^A modification patterns. The results were presented in three forms: immune score, stromal score, and the ESTIMATE score; the higher the score from the immune score or stromal score, the more immune or matrix components in TME. The ESTIMATE score is the sum of the immune score and stromal score, which represents the level of the two components in TME.

### Identification of Differentially Expressed Genes (DEGs) Among Three Types of m^6^A Modified Patterns

DEGs were identified *via* the Bayesian method between different m^6^A modification patterns using the limma R package (35), the absolute value of logFC > 1 as the significance criteria. We used the ssGSEA method to score the samples based on the 23 m^6^A regulatory factors. Based on the median value of the m^6^A score, the patients were classified into a high m^6^A score group or a low m^6^A score group. The difference between the two groups was analyzed by the empirical Bayes method using the limma R package, taking the corrected p-value < 0.05, and the absolute value of logFC > 1 as the significant criteria and 647 DEGs were obtained for subsequent signal pathway analysis.

### Differential Signaling Pathway Enrichment Among the Three m^6^A Modified Modes

The enrichment of signaling pathways for the DEGs expressed relative to the different phenotypes of m^6^A were analyzed using the metascape website (36) http://metascape.org. We determined the statistically rich terms, accumulated hypergeometric p values, and enrichment factors, and used these in a filtering step. Next, according to the Kappa statistical similarity between the gene members, the remaining important terms were clustered in a hierarchical structure, and the kappa score of 0.3 was used as a threshold to convert the terms to clusters (36). We selected the genes with the best p-value in each cluster as its representative term and displayed them in the heatmap. The databases for the KEGG pathway, GO biological processes, Reactor gene sets, Canonical pathways, and the CORE resources were used for pathway and process enrichment analysis applying a p-value < 0.01, a minimum count of 3, and an enrichment factor > 1.5 (the enrichment factor is the ratio between the observed count and the chance expected count). The GSEA analysis (37) evaluates the skewness of the two distributions for the selected gene set in the gene list sorted by a specific phenotype. The analysis gene set was obtained from the hallmark gene sets provided by the Molecular Signatures Database (https://www.gsea-msigdb.org/). In this study, we used the clusterProfiler R package to implement the GSEA analysis (38).

### Construction of a Scoring System to Evaluate the Level of m^6^A Modification in Individual Samples

To quantify the m^6^A modification pattern in a single patient. Based on the mRNA expression level of the 23 identified m^6^A regulatory molecules in each sample, we constructed a scoring system called m^6^Ascore by single sample Gene Set Enrichment Analysis (ssGSEA, based on GSVA R package).

### Analysis of Copy Number Variation in Different Subtypes of Somatic Cells

For copy number variation analysis, we used the GISTIC.2 to identify significantly amplified or missing parts of the genome. The burden of copy number loss or gain was calculated as the total number of genes with copy number changes at the focal and arm level.

### Relationship Between Methylation of m^6^A and the Core Biological Pathways Impacted Due to Bladder Cancer

We analyze a set of gene sets for storing genes related to certain biological processes, and further, to reveal the association between the m^6^A gene signature and some related biological pathways, including: (1) the immune-checkpoint; (2) antigen processing machinery; (3) CD8 T-effector signature; (4) epithelial-mesenchymal transition (EMT) markers including EMT1, EMT2, and EMT3; (5) the angiogenesis signature; (7) pan-fibroblast TGF-β response signature (Pan-F-TBRS); (8) WNT targets; (9) DNA damage repair; (10) mismatch repair; (11) Nucleotide excision repair; (12) DNA replication; (13) and antigen processing and presentation.

The differential gene expression of the core biological pathway genes between the different m^6^A phenotypes of the IMvigor210 cohort was displayed in the form of a heatmap, including: (A) FGFR3 gene signature; (B) CD8 Teff signature; (C) antigen-processing machinery; (D) immune checkpoint signature; (E) MKI67 and cell cycle genes; (F) DNA replication-dependent histones; (G) DNA damage-repair genes; (H) TGFβ receptor and ligand; (I) F-TBRS genes; (J) EMT markers; (K) angiogenesis signature.

### Cell Lines and Cell Culture

The human bladder cancer cell lines were obtained from the Oncology Institute of Central South University. The cell lines were incubated at 37°C under a humidified atmosphere with 5% CO2 and cultured in DMEM (Invitrogen, CA), supplemented with 10% fetal bovine serum (FBS) (GIBCO, NY), 1 mmol/L glutamine, and 1% penicillin/streptomycin.

### Cell Viability, Apoptosis, and Invasion Assays

Cell proliferation was analyzed using a commercial CCK-8 assay kit (#C0038, Beyotime). We employed fluorescence-activated Cell Sorting (FACS) to assess apoptosis with the Annexin V-FITC/PI staining kit (Mbchem). According to the manufacturer, the transwell assay was used to measure cell invasion with the 6-well insert devices (8 μm pore size; Corning Life Sciences, Bedford, MA) and Biocoat Matrigel (BD Biosciences). We counted the cells from the middle and surrounding 5 fields of view and averaged the counts.

### Western Blotting

Western blotting was performed as described previously (39). Briefly, total protein was extracted with RIPA lysis buffer and estimated using the bicinchoninic acid (BCA) Protein Assay Reagent Kit. The proteins were separated using 10% SDS-PAGE and electrophoretically transferred on a polyvinylidene fluoride (PVDF) membrane. The membranes were blocked with 5% skimmed milk and incubated overnight at 4°C with primary antibodies targeting FTO (27226-1-AP) and β-actin (1:2000, Ptgcn, 66009-1-Ig) and anti‐GAPDH (Abcam, ab125247), followed by incubation with the appropriate secondary antibodies for one hour. Positive bands were visualized using an enhanced chemiluminescence system.

### Measurement of Total m^6^A

Total m^6^A content was measured in the aliquots of 200-ng of total RNA extracted from cells using an m^6^A RNA methylation quantification kit (cat. no. P-9005; Epigentek) according to the manufacturer’s instructions.

### Statistical Analysis

The non-paired *t*-test was used to compare the normally distributed variables between the two groups; the Mann-Whitney U test (also known as the Wilcoxon rank-sum test) was used to estimate the statistical significance of the non-normal distribution variables. The Kruskal-Wallis test and a one-way ANOVA were used as nonparametric and parametric analysis methods (40). The correlation coefficient between TME infiltrating immune cells and the expression of m^6^A regulatory genes was calculated using the Pearson correlation method. We used the univariate Cox regression model to calculate the risk ratio (HR) for the m^6^A regulatory genes. The survminer R software package was used to determine the cut-off point for the correlation between the m^6^A scores and patient survival in each data set subgroup. The “surv-cut point” function of the maximum rank statistic was used to double score the m^6^A score by repeatedly testing all possible cut-off points, and further, the patients were classified into “high” and “low” subgroups, according to the maximum selected log-rank statistic, to reduce the batch effect in the calculations. The Kaplan-Meier method was used to plot the survival curve for prognosis analysis, and a log-rank test was used to determine the significance of the difference. The waterfall diagram of the maftools software package was used to show the mutations in the 23 m^6^A regulatory factors in the TCGA-BLCA cohort. The thermograms of the 23 mutations for the m^6^A regulatory factors were plotted. All statistical P-values were bilateral, and P < 0.05 was considered to be statistically significant.

## Results

### Survey of 23 m^6^A Regulatory Factors in Bladder Cancer

**Figure 1:**
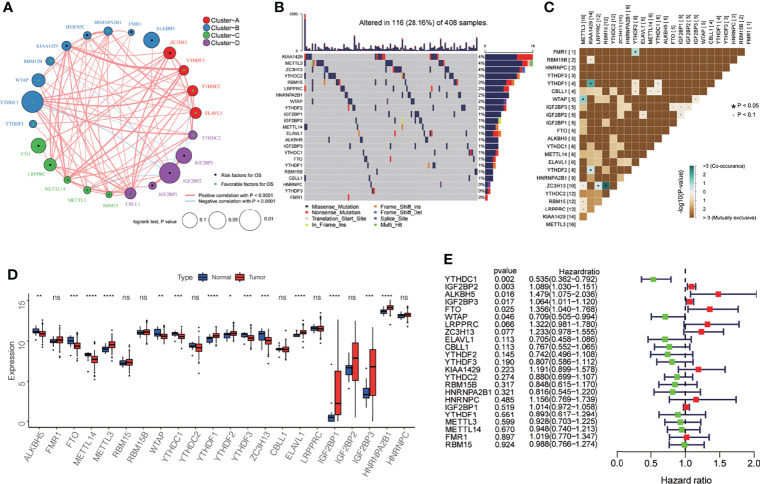
Genetic and expression characteristics of the 23 m^6^A regulatory genes in bladder cancer. **(A)** Depending on the correlation among the 23 m^6^A regulatory genes, these were divided into four groups: Cluster A, red; Cluster B, blue; Cluster C, green; Cluster D, purple. The dot in the middle of each circle represents the influence of the m^6^A regulator on OS, the favorable factors for OS are shown in green, and the risk factors are shown in black. The size of each circle represents the log-rank test p-value of the influence of the m^6^A regulator on OS (expressed as log_10_ value). The line connecting the two m^6^A regulatory factors represents the interaction between the factors; the red connection represents the positive correlation, and the light blue represents the negative correlation between the two factors. **(B)** Waterfall diagram showing 23 mutations of m^6^A regulator in bladder cancer. Each column represents the patient, each color represents the mutation type, and gray indicates that there is no mutation in the gene in the sample. The bar graph above shows the TMB, and the number on the right shows the mutation frequency of each regulator. **(C)** The mutation mode of 23 m^6^A regulatory factors; azure represents co-occurring mutations, brown represents mutually exclusive mutations, * P < 0.05, · P < 0.1. **(D)** The expression levels of 23 m^6^A regulatory factors in cancer and normal tissues. Tumor, red; normal, blue. The top and bottom of the box indicate the quartile range of the value, the lines in the box represent the median value, and the black dot represents the abnormal value. *P < 0.05; **P < 0.01; ***P < 0.001; ****P < 0.0001; ns P > 0.05. **(E)** The forest map shows the univariate Cox analysis results for the impact of 23 m^6^A regulatory factors on the OS of bladder cancer patients. Red represents risk factors, and the green represents protective factors.

In this study, we analyzed 23 m^6^A regulatory factors related to bladder cancer, including 2 erasers (*ALKBH5*, *FTO*), 13 readers (*FMR1*, *HNRNPA2B1*, *HNRNPC*, *IGF2BP1*, *IGF2BP2*, *IGF2BP3*, *YTHDC1*, *YTHDC2*, *YTHDF1*, *YTHDF2*, *YTHDF3*, *ELAVL1*, *LRPPRC*), and 8 writers (*KIAA1429*, *METTL14*, *METTL3*, *RBM15*, *RBM15B*, *WTAP*, *ZC3H13*, *CBLL1*). We first mapped the network for the 23 m^6^A regulatory factors in bladder cancer (**Figure 1A**), and examined the interactions and their significance for the prognosis of bladder cancer patients. We found that there were significant correlations between the expression of m^6^A regulatory factors in writers, erasers, and readers, and most of the correlations were positive (**Supplementary Table 1**). Based on these correlations, the 23 m^6^A regulatory factors were divided into four categories (Clusters A-D). **Figure 1B** summarizes the somatic mutation frequency of the 23 m^6^A regulators. In 116 out of 412 patient samples, a mutation in m^6^A regulators was observed, with a frequency of 28.16%. Most of the mutations were missense mutations, and the regulators *KIAA1429*, *METTL3*, and *ZC3H13* had the highest mutation frequency (4%), while *HNRNPC*, *YTHDF3*, and *FMR1* did not harbor any mutations. Subsequently, we found that *FMR1* and *YTHDF2*, *YTHDF1* and *KIAA1429*, *YTHDF2* and *KIAA1429*, *WTAP*, and *METTL3*, *ZC3H13* and *LRPPRC*, and *ZC3H13* and *YTHDC2* had significant symbiotic relationships (**Figure 1C**, P < 0.05). The differential expression analysis for the 23 m^6^A regulatory factors in bladder cancer and its adjacent tissues is shown in **Figure 1D**. The expression of *ALKBH5*, *FTO*, *METTL14*, *WTAP*, *YTHDC1*, *YTHDF3*, and *ZC3H13* in adjacent cancer tissues was significantly higher than that in cancer tissues, while the expression of *METTL3*, *YTHDF1*, *YTHDF2*, *ELAVL1*, *IGF2BP1*, *IGF2BP3*, and *HNRNPA2B1* in cancer tissues were significantly higher than those in non-cancerous tissues. However, due to the small sample size of the paracancerous tissues, this result needs further verification. A univariate Cox regression analysis was performed to study the effect of the 23 m^6^A regulatory factors on the overall survival (OS) of bladder cancer patients (**Figure 1E**). It was observed that six m^6^A regulatory factors had a significant effect on the prognosis for bladder cancer patients. *YTHDC1* and *WTAP* may be protective factors (HR < 1, P < 0.05), while *IGF2BP2*, *ALKBH5*, *IGF2BP3*, and *FTO* are risk factors for bladder cancer (HR > 1, P < 0.05).

### Three Different m^6^A Modification Patterns Defined by the 23 m^6^A Regulatory Factors

**Figure 2:**
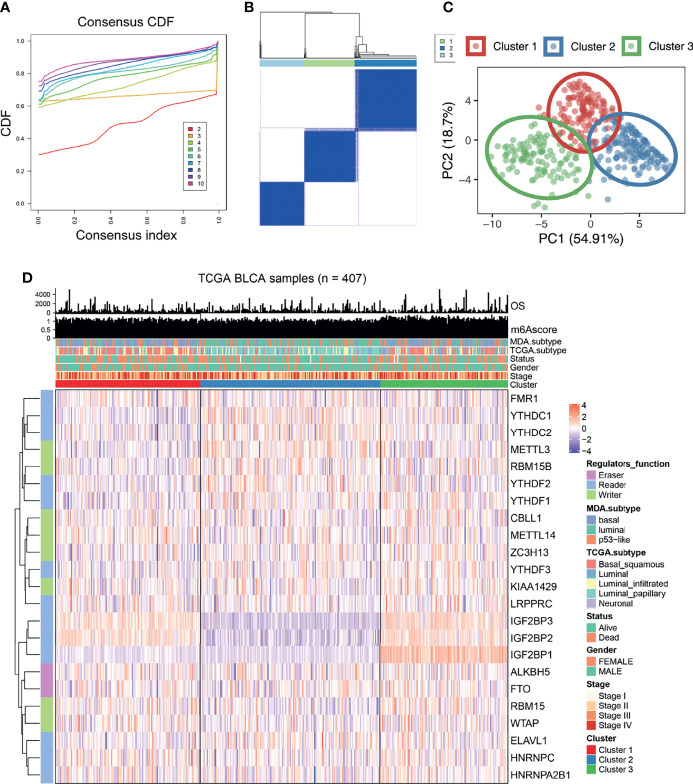
The expression of 23 kinds of m^6^A regulatory factors identified three different methylation patterns. **(A, B)** According to the expression of 23 m^6^A regulatory factors, we classified 407 bladder cancer patients, and 3 was the optimal cluster number. **(C)** Principal component analysis showed that that all patients could be well classified into three subtypes. **(D)** Heatmap display showing the expression of 23 m^6^A regulatory factors among three different m^6^A modification patterns. The relationship between different m^6^A modification patterns and clinical features such as OS, m^6^Ascore, MDA typing, TCGA typing, status, gender, stage, etc.

**Figure 3:**
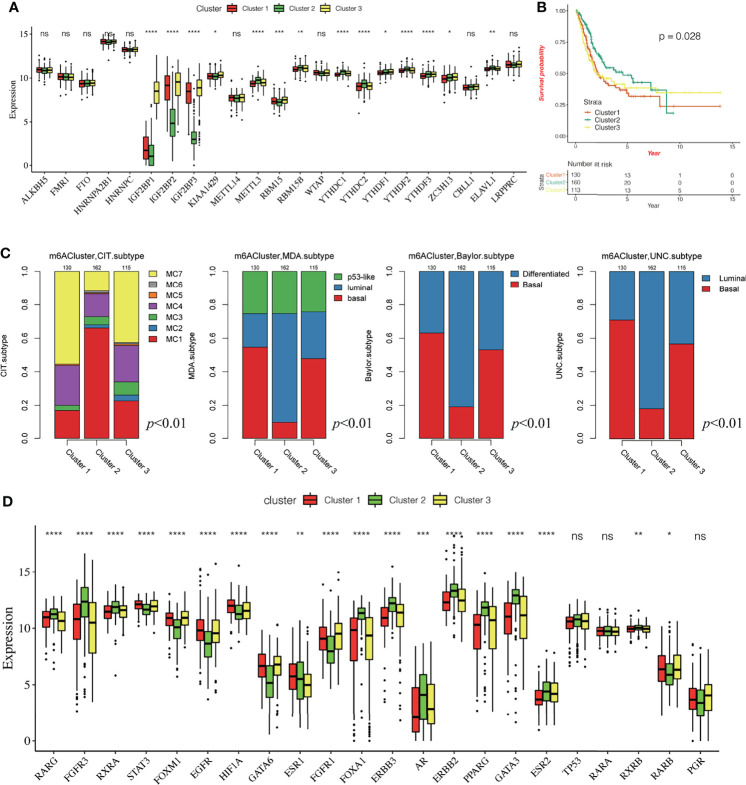
Characteristics of three different methylation patterns of m^6^A. **(A)** The expression of 23 m^6^A regulatory factors varied among the three different m^6^A modification patterns. The upper and lower end of the box indicates the quartile range of the value, the lines in the box represents the median value, the black dot represents the abnormal value, and the asterisk represents the statistical p-value, *P < 0.05; **P < 0.01; **P < 0.001, ***P < 0.0001, ****P < 0.0001. **(B)** The KM curve shows the difference in the effects of the three m^6^A modification patterns on OS. **(C)** Comparative analysis of three different m^6^A modification patterns and molecular typing of bladder cancer, including Bayol classification, MDA typing, CIT Curie typing, and UNC typing. **(D)** The upper and lower ends of the box represent the quartile range of values, the lines in the box represent the median value, and the black dots represent the abnormal values. The asterisk represents the statistical p-value, *P < 0.05; **P < 0.01; *P < 0.001, ***P < 0.0001, ****P < 0.0001, ns P > 0.05.

Based on the gene expression of the 23 m^6^A regulatory factors, 407 patients with bladder cancer were classified using the R-package ConsensusClusterPlus. Our analysis showed that 3 clusters were optimal (**Figures 2A, B** and **Supplementary Figure 1A–D**) and the principal component analysis showed that all patients could be well classified into three subtypes (**Figure 2C**), and we identified three different m^6^A modification patterns in 407 patients with bladder cancer, which we named Cluster 1-3. We plotted a heatmap (**Figure 2D**) and boxplot (**Figure 3A**) for the expression of the 23 m^6^A regulatory factors in Cluster 1-3 and found that the expression of *IGF2 BP1*, *IGF2BP2*, *IGF2BP3*, *KIAA1429*, and *RBM15* in Cluster 2 was lower than in the other clusters (P < 0.01), while the expression of *METTL3*, *RBM15B*, *YTHDC1*, *YTHDC2*, *YTHDF1*, *YTHDF2*, *YTHDF3*, and *ELAVL1* was higher in Cluster 2 than the other clusters. Significantly higher overall survival was observed in the patients who had m^6^A modifications from cluster 2 (**Figure 3B**). To better understand the potential differences between the three m^6^A modification patterns, we compared them with the published molecular typing of bladder cancers, including Baylor, MDA, CIT-Curie, and UNC (**Figure 3C**). Using the CIT classification, Cluster 1 and Cluster 3 showed more of MC7 subtypes; whereas, more MC1 subtypes were seen in Cluster 2; Using the Baylor classification, Cluster 1 and Cluster 3 had more basal subtypes, and Cluster 2 contained more differentiated subtypes; based on the UNC classification, the basal subtype was more frequent in Cluster 1 and Cluster 3 than the luminal subtype, while Cluster 2 showed the opposite result. To further characterize the molecular differences between the RNA based subtypes, we analyzed the expression of 23 regulatory genes associated with bladder cancer (41–47), including the steroid hormone receptors ESR1/2, AR, and PGR, the nuclear receptors PPARG, three RARs (A/B/G), and three RXRs (A/B/G), the receptor tyrosine kinases ERBB2/3 and FGFR1/3, and the transcription factors FOXA1, FOXM1, GATA3/6, HIF1A, KLF4, STAT3, and TP63, We found significant differences in the expression of most regulators between Cluster 2 and the other two m^6^A subtypes. The expression of *RARG*, *FGFR3*, *RXR3*, *FOXA1*, *ERBB3*, *AR*, *ERBB2*, *PPARG*, *GATA3*, *ESER2*, and *RXRB* in Cluster 2 was significantly higher than that in Cluster 1 and Cluster 3, while the opposite relation was found for *STAT3*, *FOXM1*, *EGFR*, *HIF1A*, *GATA6*, *FGFR1*, and *RARB* (**Figure 3D**). Further, we identified differences in the mutation of 20 genes in the three m^6^A modification patterns as shown in the waterfall diagram (**Supplementary Figure 1E**). and we analyzed the difference in Copy number alterations across three m^6^A modification patterns. (**Supplementary Figures 2**, **3**).

### Difference in Immune Cell Infiltration Characteristics in the Tumor Immune Microenvironment for Different m^6^A Modification Patterns

**Figure 4:**
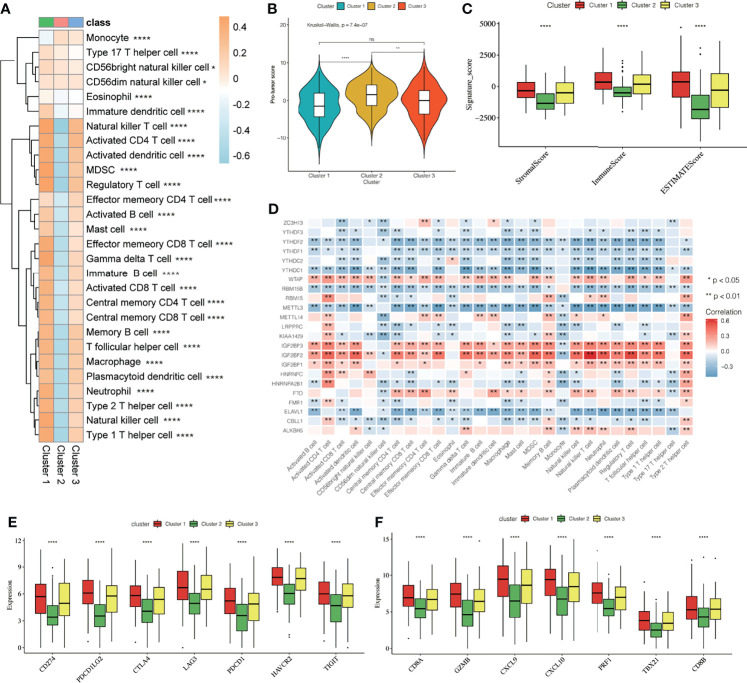
Effect of m^6^A modification on immune cell infiltration and immune checkpoint expression in the tumor immune microenvironment. **(A)** The thermogram shows the difference in the average level of 28 kinds of immune cell infiltration among three different m^6^A modification patterns, the asterisk represents the statistical p-value (*P < 0.05; **P < 0.01; ****P < 0.0001). **(B)** The violin box chart shows the difference in Pro-tumor score, the asterisk represents the statistical p-value (*P < 0.05; **P < 0.01; ****P < 0.0001, ns P > 0.05). **(C)** The ESTIMATE algorithm estimates the ratio of immune and matrix components of each sample in TME. The histogram shows the difference in immune score, stromal score, and the ESTIMATE score among the three different m^6^A modification patterns. The asterisk represents the statistical p-value (*P < 0.05; **P < 0.01; ****P < 0.0001). **(D)** Thermogram showing the 23 m^6^A regulatory factors that were correlated with 28 kinds of immune cells. The darker color indicates a higher correlation. The asterisk represents the statistical p-value (*P < 0.05, **P < 0.01). **(E, F)** The Box plot shows the immune checkpoint-related gene **(E)** and the CD8^+^T cell marker gene **(F)** among the three different m^6^A modification patterns.

To reveal the role of m^6^A modification patterns in TME immunoregulation, we calculated the abundance of 28 cellular subsets in each patient sample. The heatmap shows the average difference in the infiltration level of 28 types of immune cells in the three different m^6^A modification patterns (**Figure 4A**, **Supplementary Tables 2**, **3**), We found significant differences in the characteristics of cellular infiltration in the TME; Cluster 2 is characterized by immunosuppression, and most of the infiltrating cells are immune cells. The infiltration level was significantly lower than that of cluster1 and cluster3 (**Supplementary Figure 4A**). In addition, the anti-tumor immunity score and the pro-tumor immunity score of Cluster 2 were significantly lower than that of Cluster 1 and Cluster 3 (**Supplementary Figures 4B, C**), and the pro-tumor score of Cluster 2 is significantly higher than in Cluster 1 and Cluster 3 (**Figure 4B**). The ESTIMATE algorithm can estimate the ratio of the immune and matrix components of each sample in the TME, which is presented in the form of three scores: the immune score, stromal score, and the ESTIMATE score. We found that the three scores in Cluster 2 were significantly lower than those in Cluster 1 and Cluster 3 (**Figure 4C**), suggesting that m^6^A methylation may be involved in regulating the type of TME infiltrating cells and play a central role in immune regulation. However, the mechanism for how m^6^A modification affects the immunophenotype is unclear. Therefore, we analyzed the correlation between the 23 m^6^A regulatory factors and the 28 types of immune cells (**Figure 4D**). A heatmap of the correlation showed that the m^6^A regulators were highly correlated with most of the immune cells, and *WTAP*, *IGF2BP2*, and *IGF2BP3* showed the most significant correlation with most immune cells. Further, *YTHDF2*, *YTHDC1*, *METTL3*, and *ELAVL1* showed a high negative correlation with most immune cells, indicating that these regulators may play an important role in the differentiation and recruitment of immune cells. To further explore the possible role of m^6^A related phenotypes in immunotherapy, we studied the difference in the expression of the immune checkpoint related genes *CD274*, *PDCD1LG2*, *CTLA4*, *LAG3*, *PDCD1*, *HAVCR2*, *TIGIT*, and the CD8+T cell marker genes including *CD8A*, *GZMB*, *CXCL9*, *CXCL10*, *PRF1*, *TBX21*, and *CD8B* in the three different m^6^A modification patterns (**Figure 4E, F**). Our results showed that the expression of these genes in Cluster 2 was significantly lower than that in Cluster 1 and Cluster 3 (P < 0.0001). The results suggest that m^6^A methylation affects the type of TME infiltrating cells, and Cluster 2 had a significantly lower level of immune cell infiltration related to the immune checkpoint than the other two modification patterns. The gene expression in Cluster 2 is significantly low, and we speculated that the response to immunotherapy in patients showing Cluster 2 type m^6^A modifications may be worse than that of Cluster 1 and Cluster 3.

### Enrichment of DEGs and Signaling Pathways Involved in the Different Modes of m^6^A Modification

**Figure 5:**
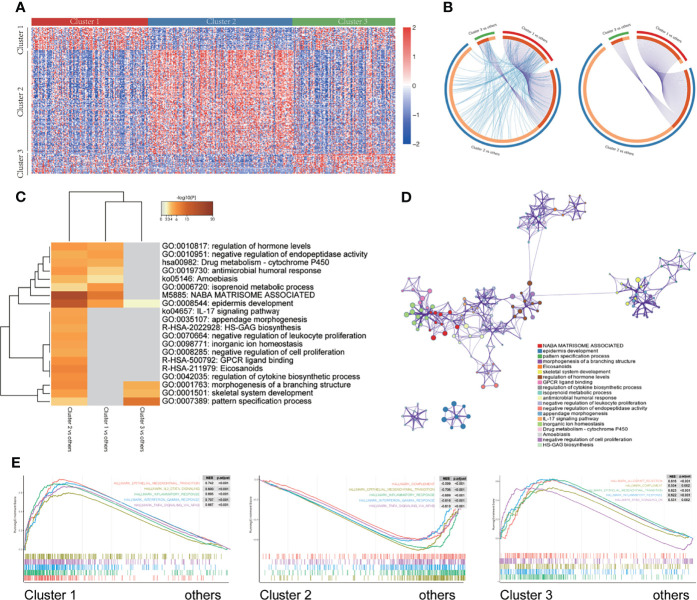
Enrichment of different gene signaling pathways in different m^6^A modification patterns. **(A)** Heatmap showing the expression of different genes in three different m^6^A modification patterns. **(B)** Circos diagram showing the overlap of differentially expressed genes by pairwise comparison of three different m^6^A modification patterns. Each arc in the outer circle represents the comparison group, and each arc in the inner circle represents a gene list. Each gene has a point on the arc. Dark orange represents the genes that appear in multiple lists, and light orange represents the only gene in the list. The purple line indicates the overlap between genes. The more purple links and the longer the dark orange arc, the higher the overlap between input gene lists, where they fall into the same ontology term (the term has to be statistically significantly enriched and with a size no larger than 100). **(C)** The thermogram shows the enrichment of gene signaling pathways with different m^6^A modification patterns. The heat map cells are colored by their p-values, and the white cells indicate that the term lacks enrichment in the corresponding gene list. **(D)** Selected representative signal paths shown in a network layout. Each signal path is represented by a circular node, whose size is directly proportional to the number of input genes in the term. The color represents the cluster identity, i.e., nodes with the same color belong to the same cluster. Terms with similarity score > 0.3 are linked by edges (the thickness of the edge represents the similarity score). The network was visualized using Cytoscape (v3.1.2). **(E)** GSEA of the marker gene set downloaded from the MSigDB database. All transcripts were sequenced according to the multiple variations (log_2_) obtained using the different analyses among three different m^6^A modification patterns. GSEA analysis evaluated the skewness of the two distributions of the selected gene set in the sequenced gene list.

To reveal the biological differences among the three different m^6^A modification patterns, we undertook expression profiling of the patient tissue samples and identified 403 DEGs (**Figure 5A**). The Circos diagram shows the overlap of the DEGs obtained by pairwise comparison of the three different m^6^A modification patterns. Cluster 2 had more DEGs than did Cluster 1, and there are more common genes in the list of DEGs obtained for Cluster 1 than that obtained for Cluster 2, and between Cluster 3 and Cluster 2 (**Figure 5B**). In **Figure 5B**, the blue lines connect genes belonging to the same ontology term showing the number of functional overlaps between different groups.

We used Metascape (36) to analyze the enrichment of signaling pathways between the different m^6^A modification classes. The results are shown in the heatmap which is colored by p-values, and the white cells indicate a lack of enrichment of the term in the corresponding gene list (**Figures 5C, D**, and **Supplementary Figures 5A, B**). Additionally, a GSEA was performed for further signaling pathway enrichment analysis (**Figure 5E**), IL2/STAT5 signaling, EMT, inflammatory response, interferon-gamma response, and TNFα signaling *via* NFκB were enriched in Cluster 1. Allograft rejection, complement, EMT transition, inflammatory response, kras signaling are enriched in Cluster 3. These signaling pathways involve different core biological processes, most of which are related to the regulation of immunity, and some of them have been confirmed to have a correlation in immunotherapy (48–50), which provides a basis for further exploration of the effect of m^6^A modifications on the immunophenotype.

### The m^6^A Score Is an Important Prognostic Biomarker and Predictor of Bladder Cancer

**Figure 6:**
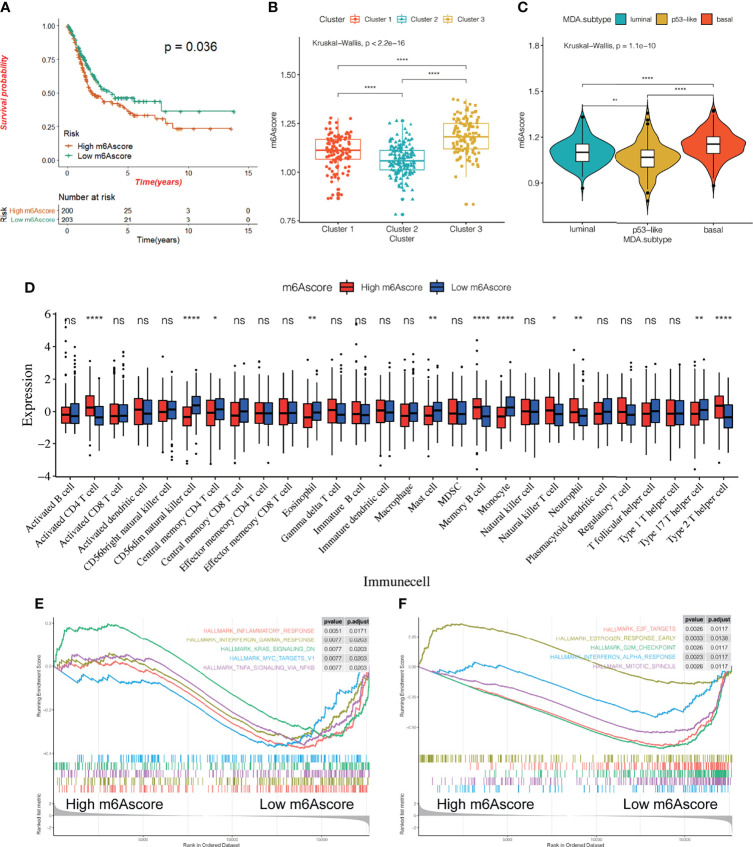
Relationship between m^6^Ascore and the characteristics of bladder cancer. **(A)** KM survival curve showing the effect of m^6^Ascore on OS in the TCGA-BLCA cohort. **(B, C)** The difference of m^6^A score among three different m^6^A modification patterns **(B)** and MDA typing **(C)**. The asterisk represents the statistical p-value (*P < 0.05; **P < 0.01; ****P < 0.0001). **(D)** According to the expression of the m^6^Ascore,407 patients were classified into a high m^6^Ascore group and a low m^6^Ascore group. The histogram shows the expression difference of 28 kinds of immune cells between the two groups. **(E, F)** All transcripts were sequenced according to the multiple variations (log_2_) obtained by the two groups in the analysis. GSEA analysis evaluated the skewness of the two distributions of the selected gene set in the sequenced gene list.

We constructed and evaluated a scoring system called the m^6^Ascore to quantify the level of m^6^A modification in each patient sample by ssGSEA, taking into account the individual differences and complexity of the m^6^A modification. First, we conducted a survival analysis using the m^6^Ascore and found that patients with high m^6^Ascore had a poor prognosis (p = 0.036, **Figure 6A**). There were also significant differences in the m^6^Ascore among the three different m^6^A modification patterns, with the lowest and highest m^6^Ascores being assigned to Cluster 3 and Cluster 2, respectively (**Figure 6B**). Next, we analyzed the differences in m^6^Ascore in the MDA cancer subtype and found the highest score for the basal subtype, followed by the luminal subtype, with the lowest expression level observed in the p53-like subtype (**Figure 6C**). To explore the potential biological relevance of m^6^Ascore, we classified 407 patients into a high m^6^Ascore group and a low m^6^Ascore group based on the expression level of the 23 m^6^A regulators. We first examined the difference in the composition of immune cells between the two patient groups (**Figure 6D**). Our results showed that CD56^dim^ natural killer cells, central memory CD4T cells, eosinophils, mast cells, monocytes, and type 17 T helper cells showed a significantly low enrichment in the high m^6^Ascore group. Further, the activated CD4^+^ T cells, memory B cells, natural killer T cells, neutrophils, and type 2 T helper cells showed highly significant enrichment in the low m^6^Ascore group. We analyzed the DEGs, and additionally examined the signaling pathways showing enrichment *via* GO and KEGG pathway analysis between the two groups. The pathways showing enrichment for molecular function (MF), cellular component (CC), and biological process (BP) *via* GO analysis were examined. Signaling pathways enriched for BP include the xenobiotic metabolic process, hormone metabolic process, epidermis development, etc.; pathways enriched for CC included the apical part of the cell, apical plasma membrane, cornified envelope, etc.; and pathways enriched for MF include monooxygenase activity, serine-type endopeptidase activity, and other signaling pathways (**Supplementary Figures 5C–E**). A KEGG pathway analysis showed that the DEGs were mainly enriched in glutathione metabolism, ether lipid metabolism, and other signaling pathways (**Supplementary Figure 5F**). A GSEA analysis (**Figures 6E, F**) showed that pathways for the inflammatory response, interferon-gamma response, myc targets V1, TNFα signaling *via* NFκB, and other genes were concentrated in the high m^6^Ascore group. These results strongly suggest a significant correlation between a high m^6^Ascore and immune activation. The results also demonstrate the central role of m^6^A methylation in different biological processes in bladder cancer, including different aspects of immune regulation. The m^6^Ascore may be used to evaluate the characteristics of immune cell infiltration in bladder cancer tissue to predict the clinical response of patients to immunotherapy. Thus, the m^6^Ascore may have the potential to be used as an independent prognostic biomarker to predict the OS rate of patients, guiding more effective clinical practice.

### Effect of m^6^A Methylation Modification on Anti-PD-L1 Immunotherapy for Bladder Cancer

**Figure 7:**
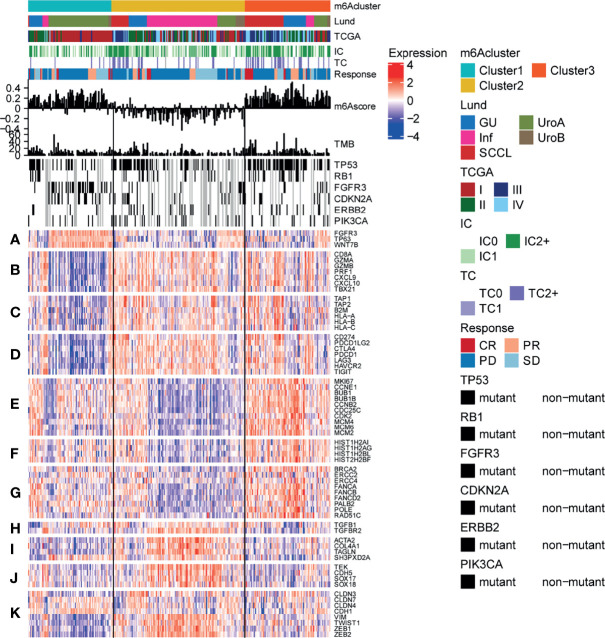
Characteristics of m^6^A modification in anti-PD-L1 immunotherapy cohort of bladder cancer. Heatmap representing all patients evaluated for anti-PD-L1 response, First, the sequence was based on the modified mode of m^6^A, next by the molecular subtype, PD-L1 expression on cells (IC), PD-L1 expression on cells (TC), and the reaction to atezolizumab. The m^6^Ascore and TMB levels of each patient are shown, and the mutation status of several genes of interest (black, mutation; gray, patients without mutation data) are shown. In addition, the lines of the heat map show the expression of the genes of interest (z-score), which are divided into the following biological classes and/or pathways: **(A)** FGFR3 gene signature; **(B)** CD8 Teff signature; **(C)** antigen-processing machinery; **(D)** immune checkpoint signature; **(E)** MKI67 and cell cycle genes; **(F)** DNA replication-dependent histones; **(G)** DNA damage-repair genes; **(H)** TGFβ receptor and ligand; **(I)** F-TBRS genes; **(J)** EMT markers; **(K)** angiogenesis signature.

**Figure 8:**
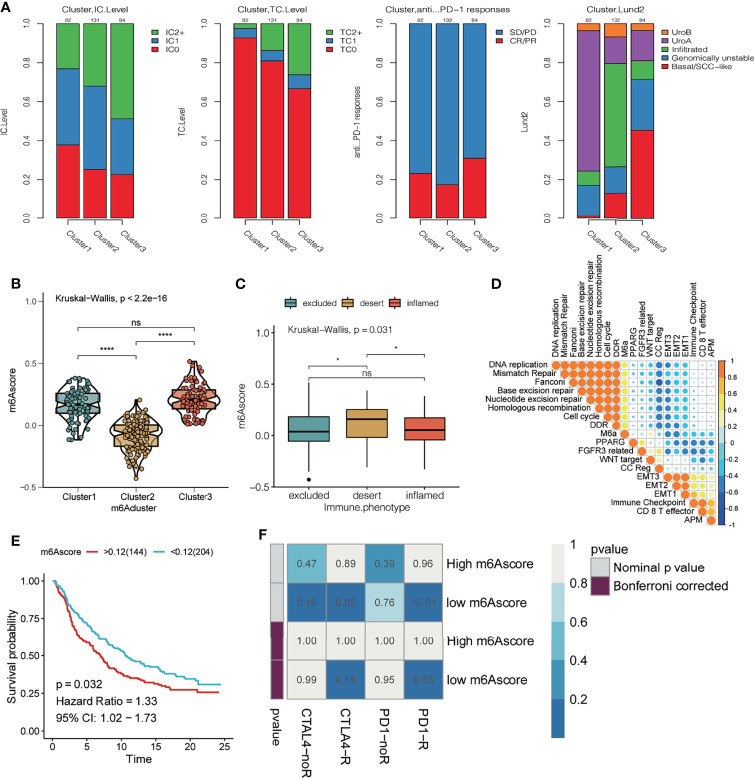
Effect of m^6^A modification mode on the treatment of bladder cancer with PD-L1. **(A)** From left to right, the stacking histogram shows the relationship between the three kinds of m^6^A modification patterns and PD-L1 expression on immune cells (IC), PD-L1 expression on tumor cells (TC), response to atezolizumab, and Lund typing. **(B)** m^6^Ascore difference between 3 different m^6^A modification patterns. The asterisk represents the statistical p-value (*P < 0.05; ****P < 0.0001, ns P > 0.05). **(C)** The difference in m^6^Ascore among the three immunophenotypes; the asterisk represents the statistical p-value (*P < 0.05; ****P < 0.0001). **(D)** Spearman correlation was used to analyze the relationship between the m^6^Ascore and known gene characteristics in the IMvigor 210 cohort. The larger the circle, higher the correlation. **(E)** KM curve showing the effect of m^6^Ascore on OS in the IMvigor210 cohort. **(F)** Using the SubMAP algorithm, we predicted response to the anti-PD1 and anti- cytotoxic T-lymphocyte-associated protein 4 (CTLA4) immunotherapy in the groups with high and low m^6^A score. High IC score group may respond better to the PD-1 treatment (Bonferroni-corrected *P* = 0.03).

To explore the potential effect and mechanism of m^6^A methylation on immunotherapy for bladder cancer, we obtained data for the IMvigor210 cohort, which is an open-label, multicenter, single-arm phase II clinical study to evaluate the safety and efficacy of Tecentriq, an anti-PD-L1 agent, in patients with advanced urothelial cancer (**Supplementary Table 4**). Patients who responded in whole or in part were classified as responders and were compared with non-responders who showed the incidence of stable or progressive disease. We performed an unsupervised clustering using data for mRNA expression for the 23 m^6^A regulatory factors from all patients. We identified three different m^6^A modification patterns and calculate the m^6^Ascore for subsequent analysis. Additionally, we performed a combined heatmap-based analysis (**Figure 7**) to find the correlation between the different m^6^A methylation modes and the Lund and TCGA molecular typing for the anti-PD-L1 based immunotherapy. Further, for the three groups of m6A modification patterns, we also analyzed the differences in PD-L1 expression in immune cells (IC) and tumor cells (TC), patients’ response to anti-PDL1 efficacy, TMB, m^6^Ascore, and the important gene mutations prevalent in bladder cancer. We also analyzed the differences in the expression of genes in important biological pathways for bladder cancer (51), including the *FGFR3* gene signature; CD8 Teff signature; antigen-processing machinery; immune checkpoint signature; MKI67 and cell cycle genes; DNA replication-dependent histones; DNA damage-repair genes; TGFβ receptor and ligand; F-TBRS genes; EMT markers; and the angiogenesis signature. In the comparison between the different m^6^A modification patterns and the TCGA and Lund classification using a heat map-based analysis, we found that most genes in Cluster 1 were classified as subtype I and the least as subtype IV. For the Lund classification, we observed that the UroA subtype had the highest proportion in Cluster 1, the Inf type has the highest proportion in Cluster 2, and the SCCL subtype was more enriched in Cluster 1. Additionally, we found that the Cluster 3 modification mode had higher levels of PD-L1 expression in immune cells and tumor cells, a higher proportion of CR/PR (**Figure 7** and **Figure 8A**). These results suggest that m^6^A methylation may be involved in the response to anti-PDL1 therapy. Additionally, among the three different m^6^A methylation patterns in bladder cancer, there were differences in gene mutations (51) for *TP53*, *RB1*, *FGFR3*, *CDKN2A*, *ERBB2*, and *PIK3CA*. Patients had a greater number of *FGFR3* and *CDKN2A* mutations in Cluster 1 than in Cluster 2 or Cluster 3, while the converse was true for *TP53* mutations. There were significant differences among the three m^6^A modification patterns in most gene sets. The expression of the *FGFR3* gene signature (*FGFR3*, *TP63*, *WNT7B*) in Cluster 1 was significantly higher than in the other two groups, while the CD8 Teff signature (*CD8A*, *GZMA*, *GZMB*, *PRF1*, *CXCL9*, *CXCL10*, *TBX21*),antigen-processing machinery (*TAP1*, *TAP2*, *B2M*, *HLA-A*, *HLA-B*, *HLA-C*), and the immune checkpoint signature (*CD274*, *PDCD1LG2*, *CTLA4*, *PDCD1*, *LAG3*, *HAVCR2*, and *TIGIT*) are less expressed in Cluster 1 than the other two modification patterns, and are highly expressed in the Cluster 3 subgroup. The CD8 Teff and the immune checkpoint signatures are often used to predict the efficacy of immunosuppression in patients with immune checkpoint suppression; a higher expression level is linked to better clinical outcomes (5, 52). Additionally, our results indicated that the expression of *MKI67* and cell cycle genes; DNA replication-dependent histones; DNA-repair genes are significantly higher in Cluster 3; TGFβ receptor and ligand; F-TBRS genes; EMT markers; angiogenesis signature show significantly increased gene expression in Cluster 2. We have observed that there are more compact types and fewer read types in Cluster 1. Compared with Lund typing, there are more SCCL types in Cluster 1, more UroA types in Cluster 1, and more infs types in Cluster 2 (**Figure 8A**). We found differences in m^6^Ascore levels among the three m^6^A modification patterns, with the lowest m^6^Ascore level found in Cluster 2, while the m^6^Ascore of Cluster 3 and Cluster 1 were the same, with no significant difference (**Figure 8B**). Differences in the m^6^Ascore were also observed in immunophenotyping, with the excluded subtype having the highest m^6^Ascore, and no significant difference in m^6^Ascore found between the inflamed and desert subtypes (**Figure 8C**).

Next, we analyzed the correlation between m^6^Ascore and biological pathway gene scores (39) in bladder cancer (**Figure 8D**). The m^6^Ascore was positively correlated with the scores for DNA replication, mismatch repair, Fanconi, base excision repair, nucleotide excision repair, homologous recombination, cell cycle, and DDR, and negatively correlated with EMT2, EMT1, immune checkpoint, CD8 T effector, APM, etc. The effect of the m^6^Ascore on OS was observed by plotting the KM curve, which indicated that a higher m^6^Ascore had a poor prognosis (P = 0.032, **Figure 8E**). Using the SubMAP algorithm, we predicted response to the anti-PD1 and anti- cytotoxic T-lymphocyte-associated protein 4 (CTLA4) immunotherapy in the groups with high and low m^6^A score. High m^6^Ascore group may respond better to the PD-1 treatment (Bonferroni-corrected *P* = 0.03)

### *FTO* Might Play an Important Role in Anti-PD-L1 Immune Checkpoint Therapy

**Figure 9:**
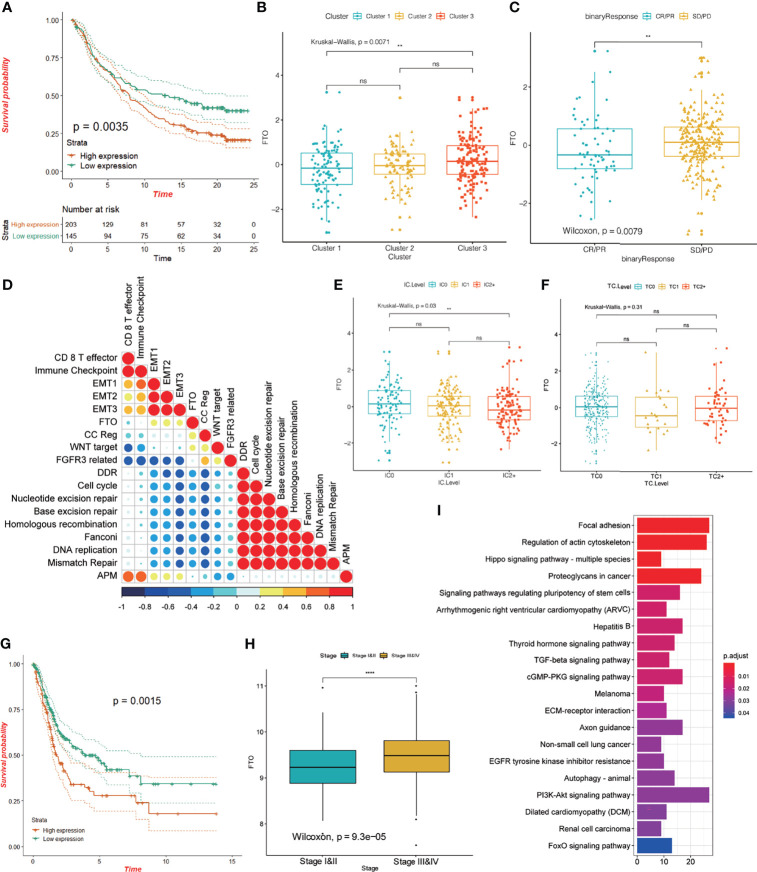
Characteristics of *FTO* in the TCGA-BLCA cohort and anti-PD-L1 treatment cohort. **(A)** KM curve showing the effect of *FTO* on OS in the IMvigor 210 cohort. **(B)** The difference in *FTO* expression among the three different m^6^A modification patterns in the IMvigor 210 cohort; the asterisk represents the statistical p-value (**P < 0.01; ****P < 0.0001, ns P > 0.05). **(C)** The difference in *FTO* expression between responders (CR/PR) and non-responders (SD/PD) in the IMvigor 210 cohort; the asterisk represents the statistical p-value (**P < 0.01; ****P < 0.0001). **(D)** Spearman’s correlation was used to analyze the correlation between *FTO* expression and known gene characteristics in the IMvigor 210 cohort. Blue, negatively correlated; orange, positively correlated; and the larger circle indicates a higher correlation. **(E, F)** The relationship between *FTO* expression and PD-L1 expression in immune cells (IC), and PD-L1 expression in tumor cells (TC). The asterisk represents the statistical p-value (**P < 0.01; ****P < 0.0001, ns P > 0.05). **(G)** KM curve showing the effect of *FTO* on OS in the TCGA-BLCA cohort. **(H)** Box plot showing the difference in *FTO* expression between stage I and II and stage III and IV; the asterisk represents the statistical p-value (**P < 0.01; ****P < 0.0001). **(I)** Pearson correlation was used to calculate the correlation between *FTO* and all other protein-coding genes, and genes with a correlation coefficient R >= 0.03, and p < 0.05 were selected as *FTO*-related genes. These genes were enriched by KEGG signaling pathway analysis to study *FTO* function.

The results from our data analysis showed that patients with high expression of *FTO* had a poor prognosis in the IMvigor210 cohort (p = 0.0035; **Figure 9A**) and there were differences in *FTO* expression in the three groups (**Figure 9B**). We also queried the predictive value of *FTO* expression in the cohort for anti-PD-L1 immunotherapy and found that there was a significant difference in *FTO* expression between non-responders and objective remission groups. Non-responsive patients showed significantly higher *FTO* expression (**Figure 9C**), suggesting that *FTO* may play an important role in the anti-PD-L1 treatment of bladder cancer. Upon further examination, *FTO* expression was positively correlated with the EMT signaling pathway and negatively correlated with the DDR/cell cycle, nucleotide excision repair, Fanconi anemia pathway, and other signaling pathways (**Figure 9D**), and additionally, was negatively correlated with the protein level of PD-L1 in immune cells (**Figure 9E**). *FTO* had no significant correlation with the total PD-L1 level in tumor cells (**Figure 9F**). The analysis of data from the TCGA database showed that the prognosis of patients with a high expression of *FTO* was worse (p = 0.0015; **Figure 9G**), and *FTO* expression was higher in patients with a high stage of bladder cancer (**Figure 9H**). Additionally, the enrichment results of *FTO*-related gene pathways showed that *FTO* may be involved in the regulation of focal adhesion, the Hippo signaling pathway, the TGF-β signaling pathway, and other signal pathways (**Figure 9I**). Previous studies have confirmed that the activation of EMT and TGF-β related pathways reduces T cell migration to tumors and weakens their tumor-killing effect (5, 51). Therefore, *FTO* may play an important role in the occurrence and development of bladder cancer, the efficacy of anti-PD-L1 immune checkpoint therapy, and might be useful for predicting the prognosis.

### *FTO* Could Be Involved in the Occurrence and Development of Bladder Cancer

**Figure 10:**
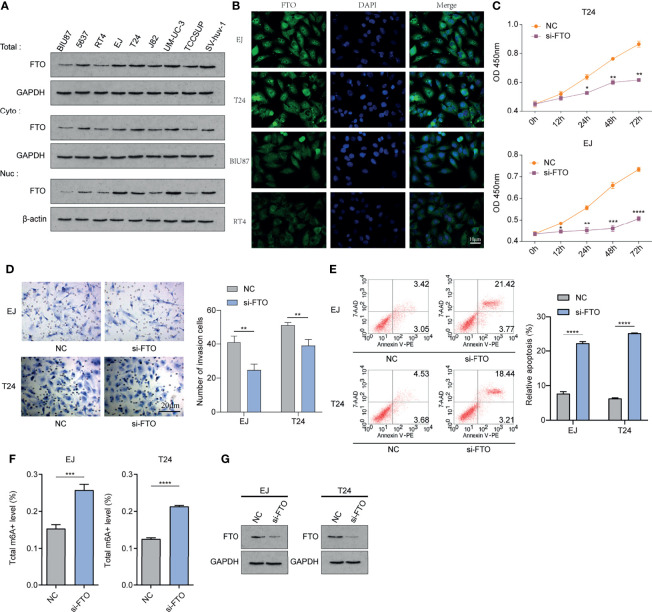
FTO could be involved in the occurrence and development of bladder cancer *in vitro.* **(A)** Western Blot method to detect nuclear, cytoplasmic, and overall expression levels in BIU87 cells, 5637 cells, T24 cells, EJ cells, RT4 cells, J82 cells, UM-UC-3 cells, TCCSUP cells, and human bladder epithelial immortalized SV-huv-1 cells. **(B)** IF immunofluorescence method to detect the cellular localization of FTO protein. **(C)** Cell Counting Kit-8(CCK-8) to detect the proliferation level of each group of cells. **(D)** Transwell method to observe the invasion level of each group of cells. **(E)** Flow cytometry method to detect apoptosis in each group. **(F)** Detection of m^6^A enzyme activity level in each group. **(G)** Western Blot method to detect the FTO expression level after siRNA knockdown. The histogram data for each group is the average of three independent replicates; bars represent mean ± SD; *P < 0.05, **P < 0.01, ***P < 0.001, ****P < 0.0001.

We examined the nuclear, cytoplasmic, and overall expression levels of FTO in nine human bladder cancer cell lines: BIU87 cells, 5637 cells, T24 cells, EJ cells, RT4 cells, J82 cells, UM-UC-3 cells, TCCSUP cells, and human bladder epithelial immortalized SV-huv-1 cells (**Figure 10A**). Using immunofluorescence two cell lines with high FTO expression (EJ and T24 cells) and two cell lines with low FTO expression (BIU87 and RT4) were identified. We found that FTO was expressed in both cytoplasm and nucleus of these cell lines (**Figure 10B**). Subsequently, we knocked down the FTO in EJ and RT4 using siRNA. CCK-8 examination revealed that cell proliferation was reduced in the si-FTO group (**Figure 10C**), and transwell assays showed reduced cell invasion in the si-FTO group (**Figure 10D**). Flow cytometry revealed increased apoptosis after the knockdown of FTO (**Figure 10E**). The level of total cellular FTO-m^6^A enzyme activity was significantly increased (**Figure 10F**), and total FTO protein was decreased in the si-FTO group (**Figure 10G**). These results confirm the critical role of FTO in bladder cancer.

**Figure 11:**
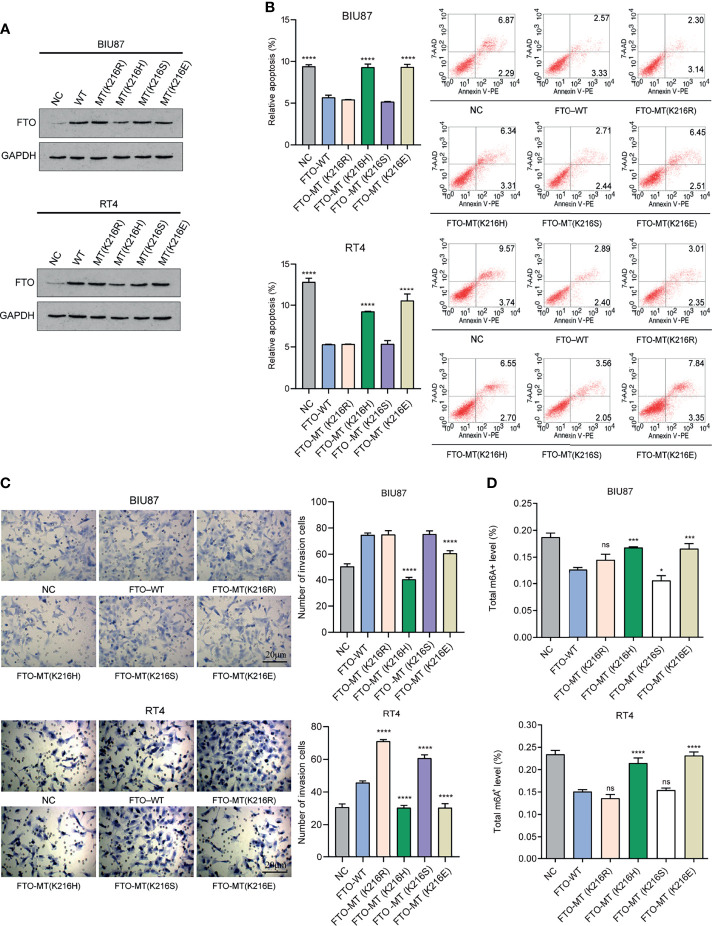
K216H and K216E are the potential important targets for regulating FTO. **(A)** Western Blot method to detect the expression level of FTO in each group. **(B)** Flow cytometry method to detect apoptosis in each group of cells. **(C)** Transwell method to observe the level of cell invasion in each group. **(D)** Detection of m^6^A enzyme activity in each group. The histogram data for each group is the average of three independent replicates; bars represent mean ± SD; *P < 0.05, ***P < 0.001, ****P < 0.0001, ns P > 0.05.

According to our previous study (53), we confirmed the existence of important modification sites K216 for FTO protein, including K216R, K216H, K216S, and K216E. We constructed plasmids and transfected them into BIU87 and RT4 cells, as well as detected the expression of FTO protein in each group by WB (Western blot) (**Figure 11A**). The apoptosis was increased in the FTO-MT (K216H) and MT (K216E) groups (**Figure 11B**), and the cell invasion was significantly reduced in the FTO-MT (K216H) and MT (K216E) groups as observed by transwell assay (**Figure 11C**), and the total FTO-m^6^A enzyme activity level was significantly increased in these two groups (**Figure 11D**). The above results suggest that K216H and K216E are the important targets for regulating FTO in the future.

## Discussion

More than 150 RNA modifications have been identified in all organisms, including 5-methylcytosine (m^5^C), N6-methyladenosine (m^6^A), and N1-methyladenosine (m^1^A), of which the m^6^A RNA methylation is considered to be the most prominent and abundant form of internal modification in eukaryotic cells (54). The study of m^6^A modification in cancer is a new field of cancer research, which may reveal a new layer of epigenetic regulation in cancer, and provide new insights into the tumorigenesis, immune response, and the molecular mechanisms underlying drug resistance during therapy. Additionally, it may lead to the development of new, effective treatments (55) through the use of effective inhibitors targeting maladjusted m^6^A regulatory factors alone (or editing targeted mutant or dysfunctional m^6^A sites through targeted transcriptomics), or in combination with other therapies. Targeting the m^6^A modification may have a strong therapeutic potential for the treatment of all types of cancers, especially those that are resistant to existing treatments (56).

Due to technical and resource constraints, most studies have focused on a single TME immune cell type or a single m^6^A. The overall infiltration characteristics of the TME mediated by the combined effects of multiple m^6^A regulators have not been widely analyzed (57, 58). The modification of m^6^A is a reversible process that is affected by various writers, erasers, and readers. In this study, we explored the effects of multiple m^6^A regulators on the infiltration of immune cells in the TME of bladder cancer, the molecular mechanisms underlying m^6^A modification in bladder carcinogenesis, the immune response, and the drug resistance of immune checkpoint inhibitors. We also provide new insights into the role of m^6^A modification patterns in TME cell infiltration and developing more effective immunotherapy strategies.

We classified patients with bladder cancer based on the expression of 23 m^6^A regulatory molecules and identified three different m^6^A modification patterns. We found that Cluster 2 showed unique specificity compared with the other two types. A quantification of 28 immune cells in the immune microenvironment of bladder cancer using the ssGSEA algorithm showed that Cluster 2 had a low level of immune cell infiltration characterized by immune inhibition, which corresponds to the immune desert phenotype. Furthermore, an analysis performed using the ESTIMATE algorithm showed that the stromal and immune scores in Cluster 2 were lower than the other two subtypes, which corroborates our previous results. The expression of immune checkpoint and CD8^+^T cell markers were lower in Cluster 2 than in the other two groups, and studies have shown that the expression of these markers is highly sensitive to immune checkpoint inhibitors (52). Thus, different m^6^A modification patterns are significantly related to immune activation, and a comprehensive evaluation of the m^6^A modification patterns will enable the understanding of the characteristics of TME cell infiltration. An analysis of DEGs between the different m^6^A modification patterns uncovered m^6^A-related signaling pathway genes. These were mainly enriched in the biological pathways related to the matrix and immune activation. In the anti-PD-L1 treatment cohort, we identified three different m^6^A modification patterns and found significant differences in gene expression and mutations in biological pathway genes associated with the three subtypes of bladder cancer. We constructed a scoring system to quantify and evaluate the m^6^A modification level in a single tumor and found that a high m^6^Ascore was related to poor prognosis in the TCGA and anti-PD-L1 cohorts. Patients with a high or low m^6^Ascore showed differences in immune cell infiltration in the tumor immune microenvironment. A GSEA signaling pathway analysis indicated that the high and low m^6^Ascore are mainly enriched in the matrix and immune-related signaling pathways. Together, these results indicate that the m^6^A score is a reliable tool for the comprehensive evaluation of m^6^A modification patterns in tumors in individuals, and to determine the mode of infiltration in the TME. The expression and gene changes for m6A regulatory factors are related to a variety of biological processes, especially the matrix and immune activation. Our findings may provide new impetus for improving the clinical response of patients with bladder cancer to immunotherapy, the identification of different immunophenotypes in bladder cancer, and promoting individualized immunotherapy.

The role of *FTO* in cancer has recently garnered increasing attention. Previous studies conducted by our team have shown that *FTO*-mediated m^6^A modification plays an important role in hepatocellular carcinoma, and the SIRT1 deacetylase can play a carcinogenic role by down-regulating *FTO*. *FTO* is an RNA demethylase that can remove the methylation of m^6^A in mRNA both *in vitro* and *in vivo* (59, 60). *FTO* has catalytic demethylation activity for both cap-m^6^Am and internal m^6^A. As the abundance of m^6^A in mRNA is much higher than that of its preferred binding target m^6^Am, the main target of *FTO* is m^6^A (61). Reports indicate that the *FTO*-mediated demethylation of cap-m^6^Am leads to mRNA degradation (62), but the evidence for this is inconsistent. PCIF1 is the cap-m^6^Am methyltransferase, which processes cap-m^6^A alone but not internal m^6^A. Reports indicate that the cap-m^6^Am added by PCIF1 does not change the level of gene expression or the stability of transcripts (63–65). Additionally, studies (66) have found that the spatial distribution of *FTO* can also play a regulatory role. The N-terminus of FTO has an NLS, which can be partially distributed in the nucleus and the cytoplasm, and the distribution of FTO is different in different cell lines, its role and regulation being affected by the environment. Yang et al. (20) have shown that the induction of *FTO* can be used as an adaptive mechanism to combat metabolic stress in melanoma cells, thus increasing their proliferation, invasion, and migration, and promoting the tumorigenesis and development of melanoma in mice. Further, the authors showed that an *FTO* knockout can increase m^6^A methylation in key oncogenic melanoma cells, including the loci for PD-1 (*PDCD1*), *CXCR4*, and *SOX10*. The inhibition of *FTO* makes melanoma cells sensitive to interferon-γ (IFN-γ) and anti-PD-1 therapy in mice, indicating that *FTO* plays an important role in promoting the occurrence of melanoma and anti-PD-1 drug resistance. The role of m^6^A involvement with *FTO* in bladder cancer had not been reported, and we found that in the TCGA and IMvigor210 cohorts, patients with high *FTO* expression had a poor prognosis, and the expression of *FTO* was higher in patients with a higher stage of bladder cancer.

In addition, we analyzed the predictive value of *FTO* expression in the cohort subjected to anti-PD-L1 immunotherapy, and found that there was a significant difference in *FTO* expression between the non-responders and the objective remission group. The level of *FTO* expression in the patients non-responsive to anti-PD-L1 therapy was significantly higher than in patients in remission. The data showed that *FTO* expression was negatively correlated with the protein level of PD-L1 in immune cells (**Figure 9E**), was positively correlated with the EMT signaling pathway, and negatively correlated with DDR, cell cycle, nucleoside exercise repair, Fanconi anemia pathway, and other signaling pathways. The enrichment of *FTO*-related gene pathways indicated that *FTO* might be involved in the regulation of focal adhesion/Hippo signaling pathway/TGF-β signaling, these results suggest that *FTO* plays an important role in bladder cancer. The combination of *FTO* targeted regulation and anti-PD-L1 blockers may have great therapeutic potential for reducing the resistance of bladder cancer to immunotherapy. Thus, this study furthers the understanding of the regulation of m^6^A modification in the tumor immune microenvironment of the bladder cancer and contributes to the development of new predictive indicators, drug combination strategies, and new immunotherapeutic strategies for cancer immunotherapy. However, its specific role and mechanism need further experimental study.

In this study, we systematically analyzed the mutation and correlation of 23 kinds of m^6^A regulatory factors in bladder cancer, and their effects on OS and immune invasion, We found three different patterns of m^6^A modification and compared them with other important molecular types of bladder cancer, such as MDA, Lund, and Baylor, the effects of the three kinds of m^6^A modification patterns on the mutation characteristics, clinicopathological characteristics, gene expression, immune cell infiltration level, and gene expression level of immune checkpoint regulators were comprehensively analyzed. Further, we investigated the effect of m^6^A modification mode on the therapeutic efficacy of bladder cancer immune checkpoint inhibitor anti-PD-L1. Our results confirmed that m^6^A methylation is involved in the process of immune cell recruitment in the TME of bladder cancer, and may affect the efficacy of anti-PD-L1 therapy. In addition, we established a method to quantify the level of m^6^A modification (m6Ascore), which we found to be an important and powerful prognostic biomarker and predictor for bladder cancer. Thus, this study furthers the understanding of the regulation of m^6^A modification in the tumor immune microenvironment of bladder cancer and contributes to the potential development of new predictive indicators, drug combination strategies, and new immunotherapeutic strategies for cancer immunotherapy.

## Conclusion

This study comprehensively recognized the role of m^6^A methylation modification on the invasion characteristics of bladder cancer immune microenvironment cells and the effect on the anti-PD-L1 treatment for bladder cancer. The difference in m^6^A modification mode is an important factor indicating the heterogeneity and complexity of the tumor microenvironment and the immunotherapy impact. The m^6^A modification mode helps decipher the molecular mechanisms underlying the immune microenvironment regulation in bladder cancer and provides new predictive indicators, possible auxiliary targets, and directions for guiding more effective immunotherapy strategies in the future.

## Data Availability Statement 

The datasets presented in this study can be found in online repositories. The names of the repository/repositories and accession number(s) can be found in the article/**Supplementary Material**.

## Ethics Statement

The experimental protocol was established according to the ethical guidelines of the Helsinki Declaration and was approved by the Human Ethics Committee of the Ethical Review Committee of the Third Xiangya Hospital of Central South University. Written informed consent was obtained from individuals or guardians of the participants.

## Author Contributions

XC and KC designed the study. XC and HD analyzed and interpreted the data. HD and XC wrote this manuscript. HD, FT, MZ, and DS conducted the experiments and edited and revised the manuscript. All authors have seen and approved the final version of the manuscript.

## Funding

This work was supported by The Science and Technology Innovation Program of Hunan Province (2020RC2066), Hunan Provincial Natural Science Foundation of China (2020JJ5335), Science and Technology Foundation of Changsha City (kq1907127), Health Commission Science Foundation of Hunan Province (20200957), Hunan Cancer Hospital Climb Plan (QH201904), The Science and Technology Innovation Program of Hunan Province (2021RC2041), and The National Science Foundation of China (81874137).

## Conflict of Interest

The authors declare that the research was conducted in the absence of any commercial or financial relationships that could be construed as a potential conflict of interest.

## Publisher’s Note

All claims expressed in this article are solely those of the authors and do not necessarily represent those of their affiliated organizations, or those of the publisher, the editors and the reviewers. Any product that may be evaluated in this article, or claim that may be made by its manufacturer, is not guaranteed or endorsed by the publisher.

## Supplementary Material

Supplementary Figure 1 Three m^6^A modification patterns were identified in the TCGA-BLCA cohort through unsupervised clustering of 23 m^6^A regulatory factors. **(A–D)** Consensus matrices of the TCGA-BLCA cohort for k = 2-5. **(E)** Waterfall diagram showing the influence of the three m^6^A modification patterns on the mutation of the top 20 genes in the TCGA-BLCA cohort. Each column represents the patient, each color represents the mutation type, and gray indicates that there is no mutation in the gene in this sample. The bar graph above shows the TMB, and the number on the right shows the mutation frequency of each gene.

Supplementary Figure 2 GISTIC analysis of foci of recurrent amplification and deletion. Detailed cytoband with focal amplification **(A)** and focal deletion **(B)** in Cluster 1, Cluster 2, and Cluster 3 generated with GISTIC 2.0 software. The q value of each locus is plotted horizontally.

Supplementary Figure 3 Copy number alterations across three m6A modification patterns. Copy number Gistic score **(A)** and Copy number profiles **(B)** for Cluster 1, Cluster 2, and Cluster 3, with gains are shown in dark red and losses in midnight blue. Gene segments are placed according to their location on chromosomes, ranging from chromosome 1 to chromosome 22.

Supplementary Figure 4 Difference of immune cell infiltration characteristics in tumor immune microenvironment for the three m^6^A modification patterns in the TCGA-BLCA cohort. **(A)** The box plot shows the difference for 28 immune cell infiltration levels in the three m^6^A modification patterns; the asterisk represents the statistical p-value (*P < 0.05; **P < 0.01; ***P < 0.001, ****P < 0.0001). **(B, C)** The differences in the antitumor immunity score and pro-tumor immunity score among three kinds of m^6^A modification patterns; the asterisk represents the statistical p-value.

Supplementary Figure 5 Signaling pathway enrichment analysis. **(A, B)** In the TCGA-BLCA cohort, there are three different m^6^A modification patterns, and different gene signaling pathways are enriched; the same enrichment network has its nodes displayed as pies. Each pie sector is proportional to the number of hits that originated from a gene list. Color code for the pie sector represents a gene list and is consistent with the colors used for the table rows in the slide. The same enrichment network has its nodes colored by p-value, as shown in the legend. Darker color indicates more statistically significant nodes (see legend for p-value ranges). **(C–E)** The enrichment analysis of Gene Ontology (GO) signaling pathways in the high and low m^6^A groups was divided into three parts: C, cellular component (CC); D, biological process (BP); E, molecular function (MF). **(F)** Enrichment analysis of KEGG signaling pathways of DEGs in high and low m^6^A groups.

Supplementary Table 1 Pearson correlation of 23 m^6^A regulatory factors

Supplementary Table 2 Immune cell marker genes

Supplementary Table 3 Infiltration levels of 28 immune cells and anti-tumor immunity score and pro-tumor immunity scores of 407 samples in the TCGA-BLCA cohort

Supplementary Table 4 IMvigor 210 cohort clinical information and important gene set score

## Abbreviations

m^6^A, N6-methyladenosine; TCGA, The Cancer Genome Atlas; GEO, Gene-Expression Omnibus; KEGG, Kyoto encyclopedia of gene and genome; GO, Gene ontology; GSEA, Gene set enrichment analysis; SsGSEA, The single-sample gene set enrichment analysis; TME, Tumor microenvironment; TMB, Tumor mutation burden; ICT, The immune checkpoint therapy; CTLA-4, Cytotoxic lymphocyte antigen-4; PD-1, Programmed cell death protein 1; PD-L1, Programmed-death ligand 1; DCs, Dendritic cells; DEGs, Differentially expressed genes; EMT, Epithelial-mesenchymal transition; ICB, Immunological checkpoint blockade; TGFb, Transforming growth factor beta
