# Supplementary material for: Identification and Validation of N6-Methyladenosine-Related Biomarkers for Bladder Cancer: Implications for Immunotherapy
Source: Front Oncol. 2022 Mar 2;12:820242. doi: 10.3389/fonc.2022.820242 (PMC8924666; doi:10.3389/fonc.2022.820242)
Supplement: Supplementary Figure 1 — Three m6A modification patterns were identified in the TCGA-BLCA cohort through unsupervised clustering of 23 m6A regulatory factors. (A–D) Consensus matrices of the TCGA-BLCA cohort for k = 2-5. (E) Waterfall diagram showing the influence of the three m6A modification patterns on the mutation of the top 20 genes in the TCGA-BLCA cohort. Each column represents the patient, each color represents the mutation type, and gray indicates that there is no mutation in the gene in this sample. The bar graph above shows the TMB, and the number on the right shows the mutation frequency of each gene. [file DataSheet_1.pdf]

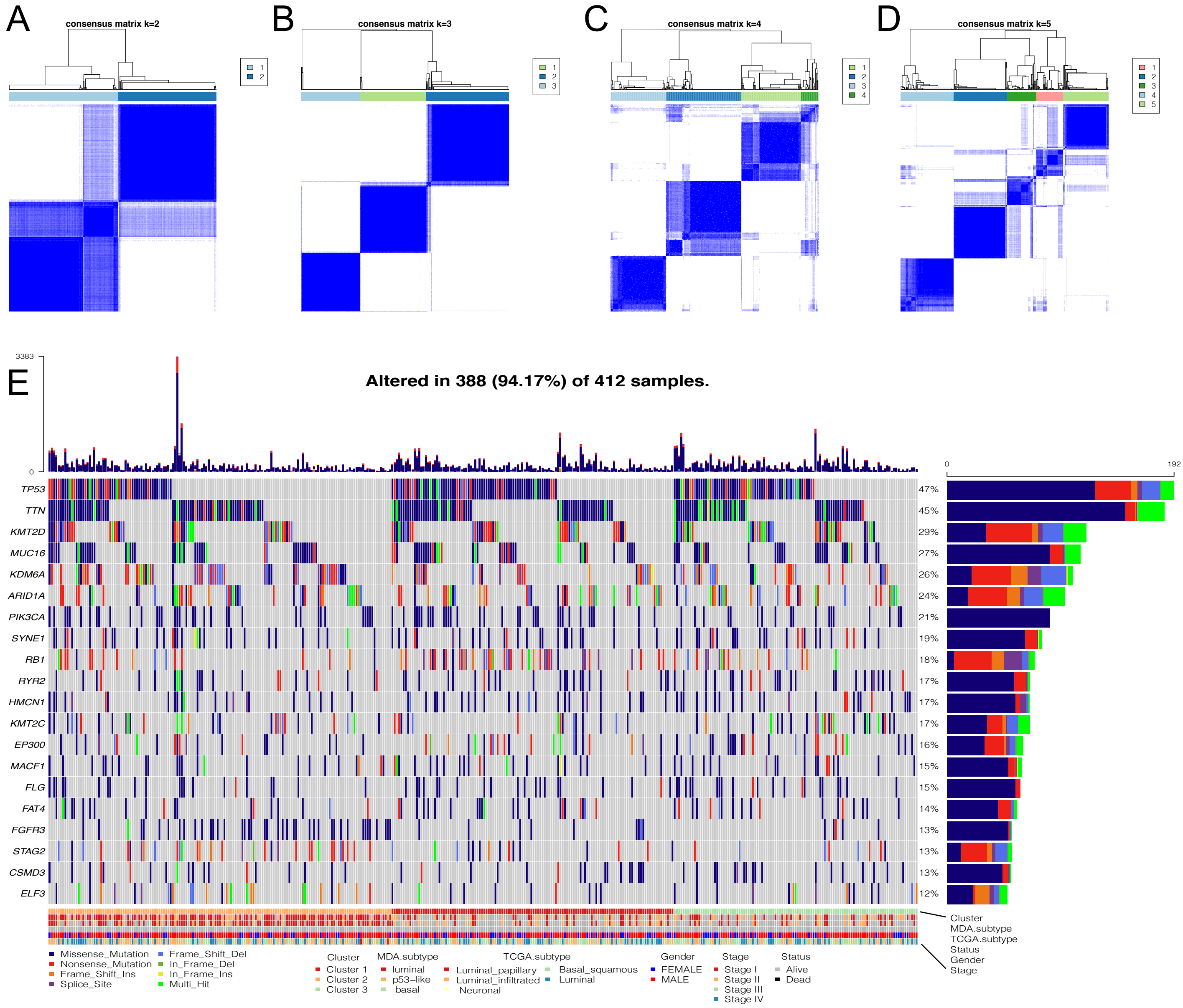
A
consensus matrix k=2
1
2
B
consensus matrix k=3
1
2
3
C
consensus matrix k=4
1
2
3
4
D
consensus matrix k=5
1
2
3
4
5
E
Altered in 388 (94.17%) of 412 samples.
3383
0
192
TP53 47%
TTN 45%
KMT2D 29%
MUC16 27%
KDM6A 26%
ARID1A 24%
PIK3CA 21%
SYNE1 19%
RB1 18%
RYR2 17%
HMCN1 17%
KMT2C 17%
EP300 16%
MACF1 15%
FLG 15%
FAT4 14%
FGFR3 13%
STAG2 13%
CSMD3 13%
ELF3 12%
Cluster
MDA.subtype
TCGA.subtype
Status
Gender
Stage
Missense_Mutation
Nonsense_Mutation
Frame_Shift_Ins
Splice_Site
Frame_Shift_Del
In_Frame_Del
In_Frame_Ins
Multi_Hit
Cluster
Cluster 1
Cluster 2
Cluster 3
MDA.subtype
luminal
p53−like
basal
TCGA.subtype
Luminal_papillary
Luminal_infiltrated
Neuronal
Basal_squamous
Luminal
Gender
FEMALE
MALE
Stage
Stage I
Stage II
Stage III
Stage IV
Status
Alive
Dead

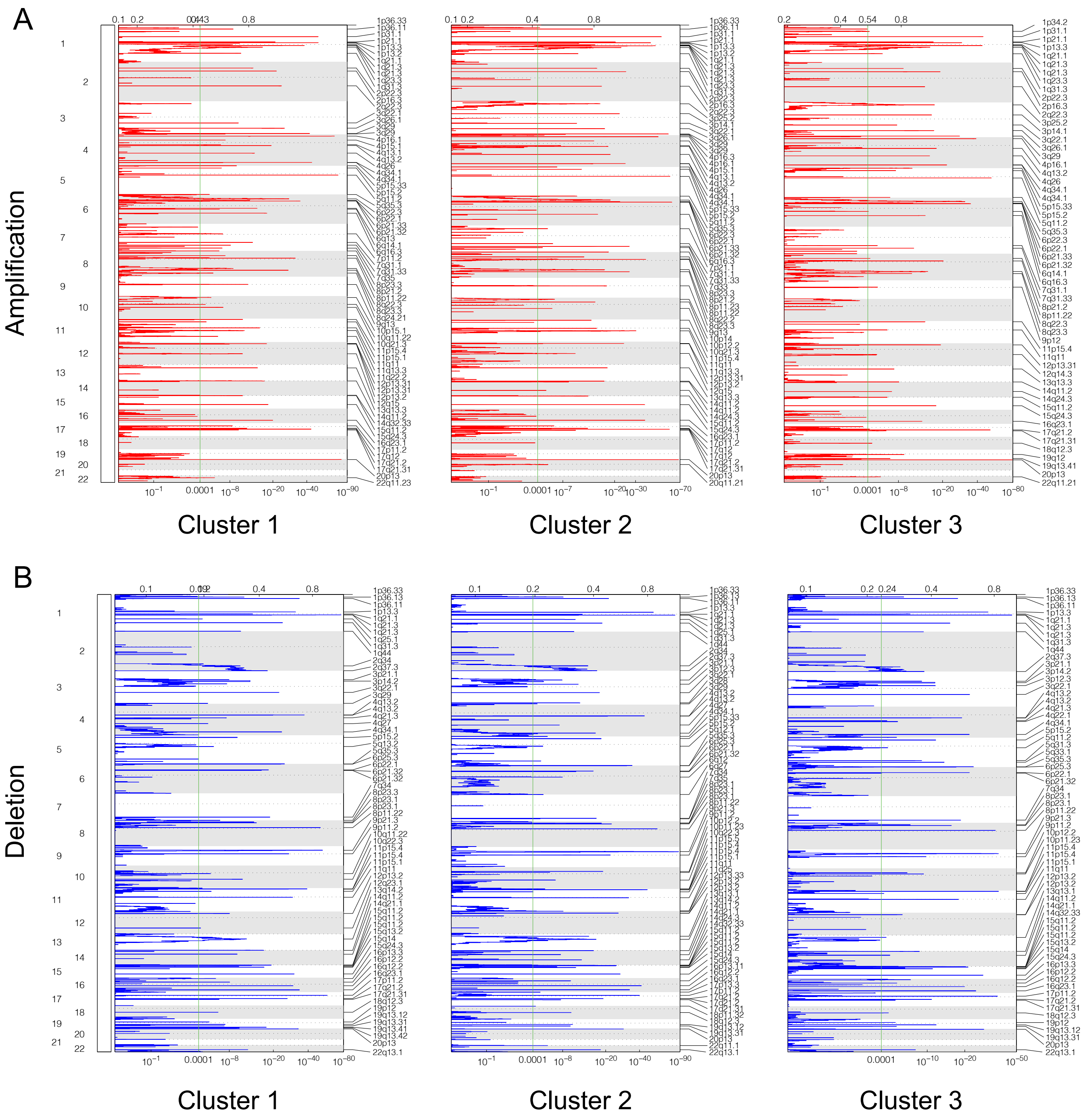

A
Amplification
Cluster 1
Cluster 2
Cluster 3
B
Deletion
Cluster 1
Cluster 2
Cluster 3

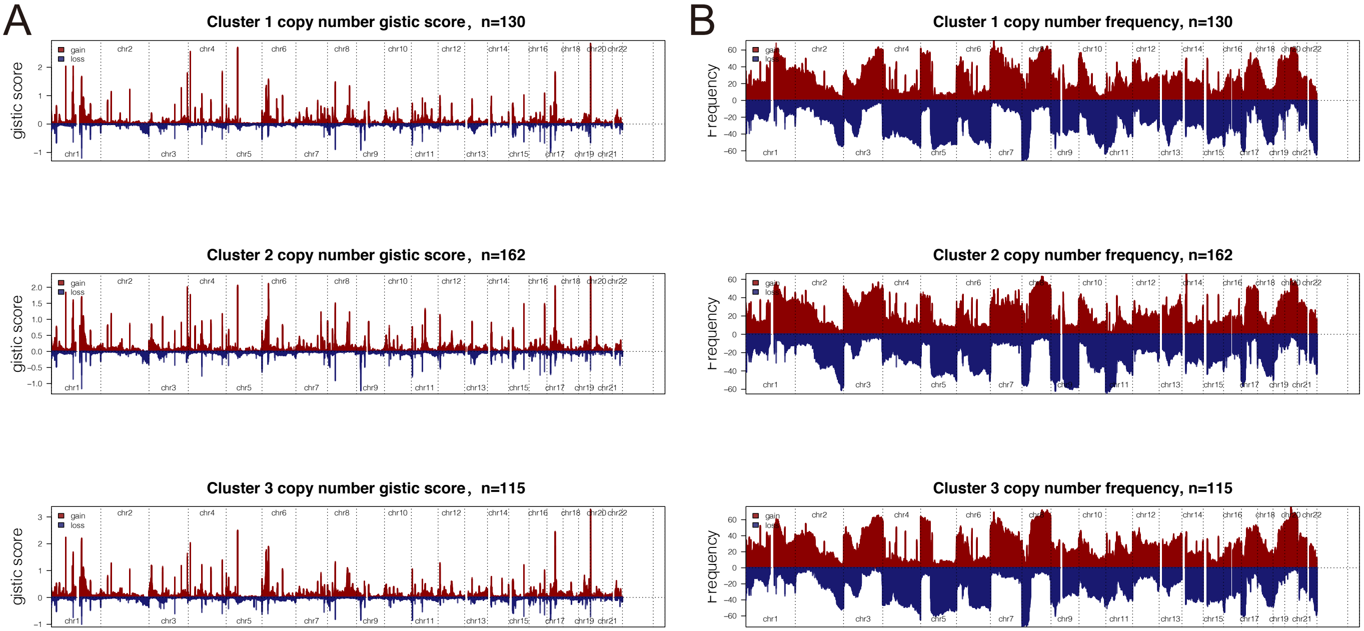

A
Cluster 1 copy number gistic score，n=130
gistic score
gain
loss
Cluster 2 copy number gistic score，n=162
gistic score
gain
loss
Cluster 3 copy number gistic score，n=115
gistic score
gain
loss
B
Cluster 1 copy number frequency, n=130
Frequency
gain
loss
Cluster 2 copy number frequency, n=162
Frequency
gain
loss
Cluster 3 copy number frequency, n=115
Frequency
gain
loss
chr1
chr2
chr3
chr4
chr5
chr6
chr7
chr8
chr9
chr10
chr11
chr12
chr13
chr14
chr15
chr16
chr17
chr18
chr19
chr20
chr21
chr22

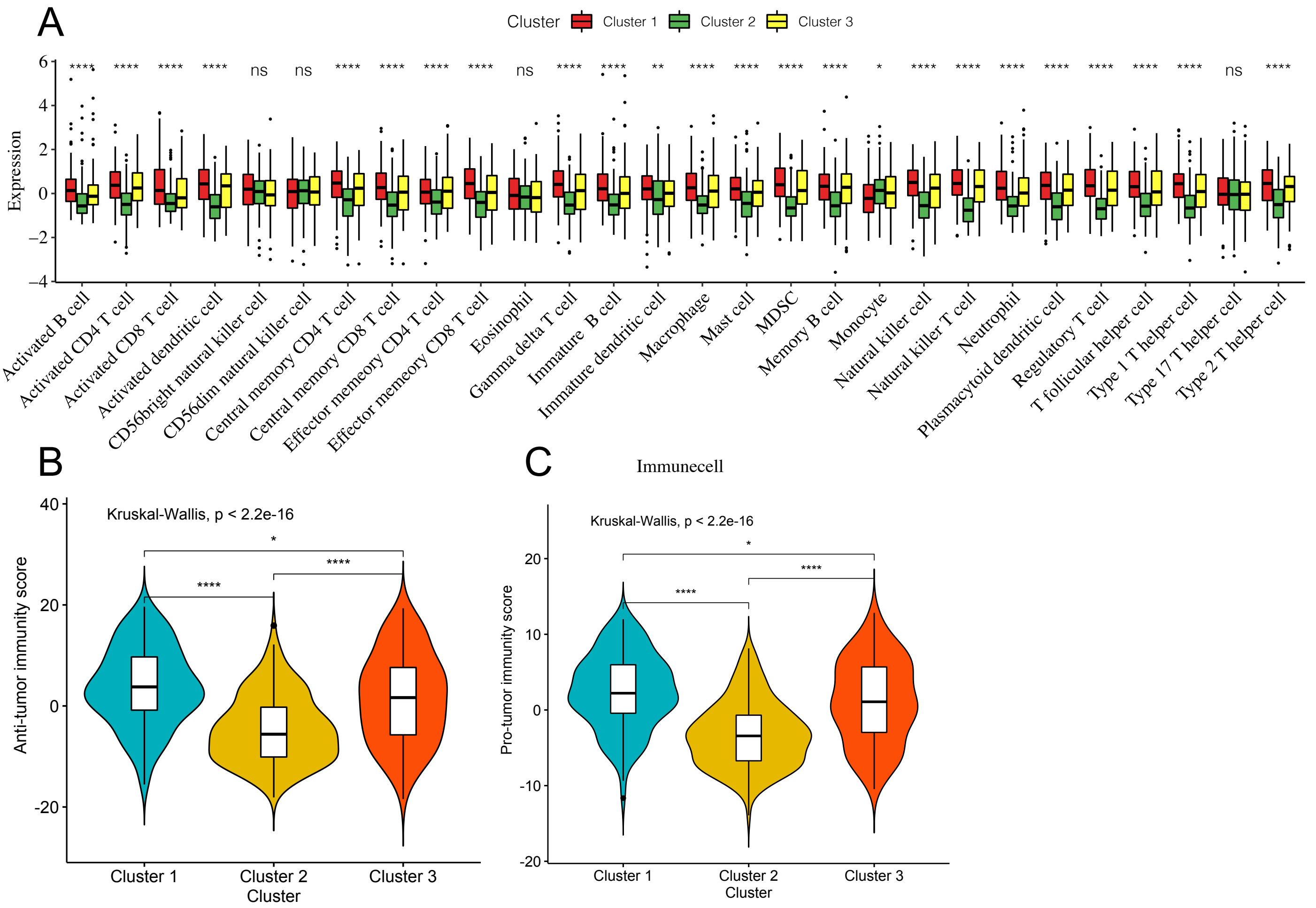
A
Cluster
Cluster 1
Cluster 2
Cluster 3
Expression
Activated B cell
Activated CD4 T cell
Activated CD8 T cell
Activated dendritic cell
CD56bright natural killer cell
CD56dim natural killer cell
Central memory CD4 T cell
Central memory CD8 T cell
Effector memeory CD4 T cell
Effector memeory CD8 T cell
Eosinophil
Gamma delta T cell
Immature B cell
Immature dendritic cell
Macrophage
Mast cell
MDSC
Memory B cell
Monocyte
Natural killer cell
Natural killer T cell
Neutrophil
Plasmacytoid dendritic cell
Regulatory T cell
T follicular helper cell
Type 1 T helper cell
Type 17 T helper cell
Type 2 T helper cell
B
Kruskal-Wallis, p < 2.2e-16
Anti-tumor immunity score
Cluster 1
Cluster 2
Cluster 3
Cluster
C
Immunecell
Kruskal-Wallis, p < 2.2e-16
Pro-tumor immunity score
Cluster 1
Cluster 2
Cluster 3
Cluster

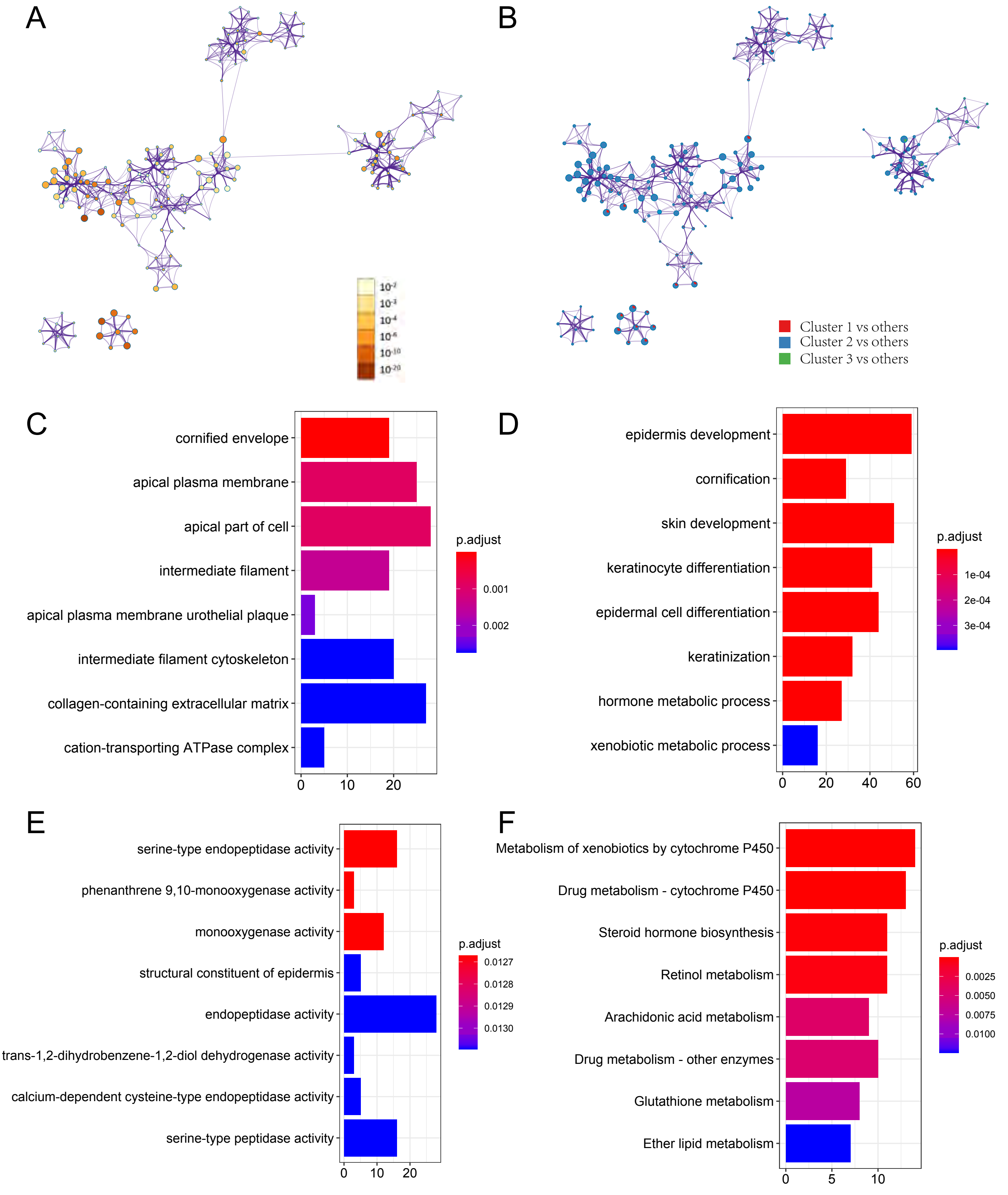
A
B
Cluster 1 vs others
Cluster 2 vs others
Cluster 3 vs others
C
cornified envelope
apical plasma membrane
apical part of cell
intermediate filament
apical plasma membrane urothelial plaque
intermediate filament cytoskeleton
collagen-containing extracellular matrix
cation-transporting ATPase complex
p.adjust
0.001
0.002
0
10
20
D
epidermis development
cornification
skin development
keratinocyte differentiation
epidermal cell differentiation
keratinization
hormone metabolic process
xenobiotic metabolic process
p.adjust
1e-04
2e-04
3e-04
0
20
40
60
E
serine-type endopeptidase activity
phenanthrene 9,10-monooxygenase activity
monooxygenase activity
structural constituent of epidermis
endopeptidase activity
trans-1,2-dihydrobenzene-1,2-diol dehydrogenase activity
calcium-dependent cysteine-type endopeptidase activity
serine-type peptidase activity
p.adjust
0.0127
0.0128
0.0129
0.0130
0
10
20
F
Metabolism of xenobiotics by cytochrome P450
Drug metabolism - cytochrome P450
Steroid hormone biosynthesis
Retinol metabolism
Arachidonic acid metabolism
Drug metabolism - other enzymes
Glutathione metabolism
Ether lipid metabolism
p.adjust
0.0025
0.0050
0.0075
0.0100
0
5
10

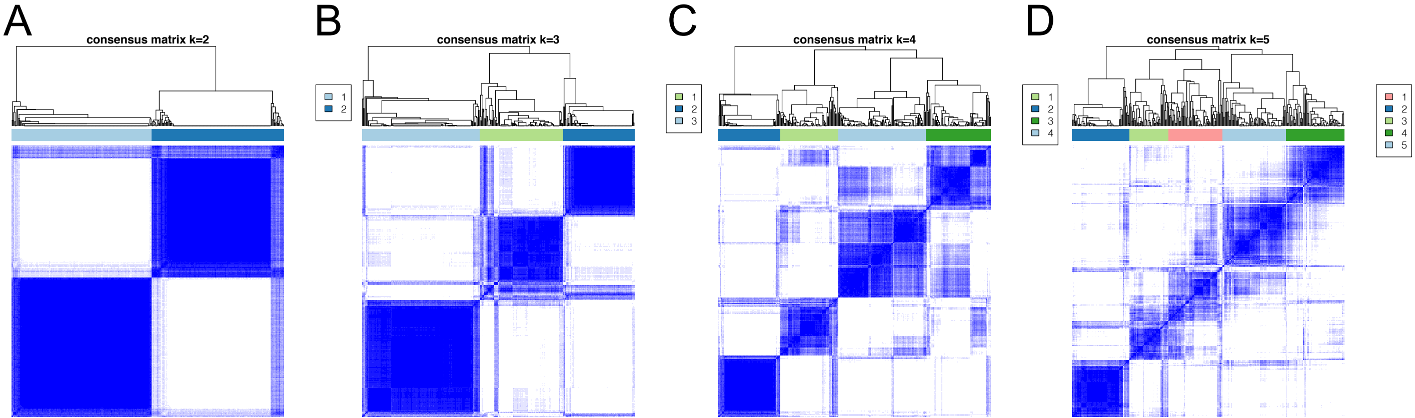
A
consensus matrix k=2
1
2
B
consensus matrix k=3
1
2
3
C
consensus matrix k=4
1
2
3
4
D
consensus matrix k=5
1
2
3
4
5

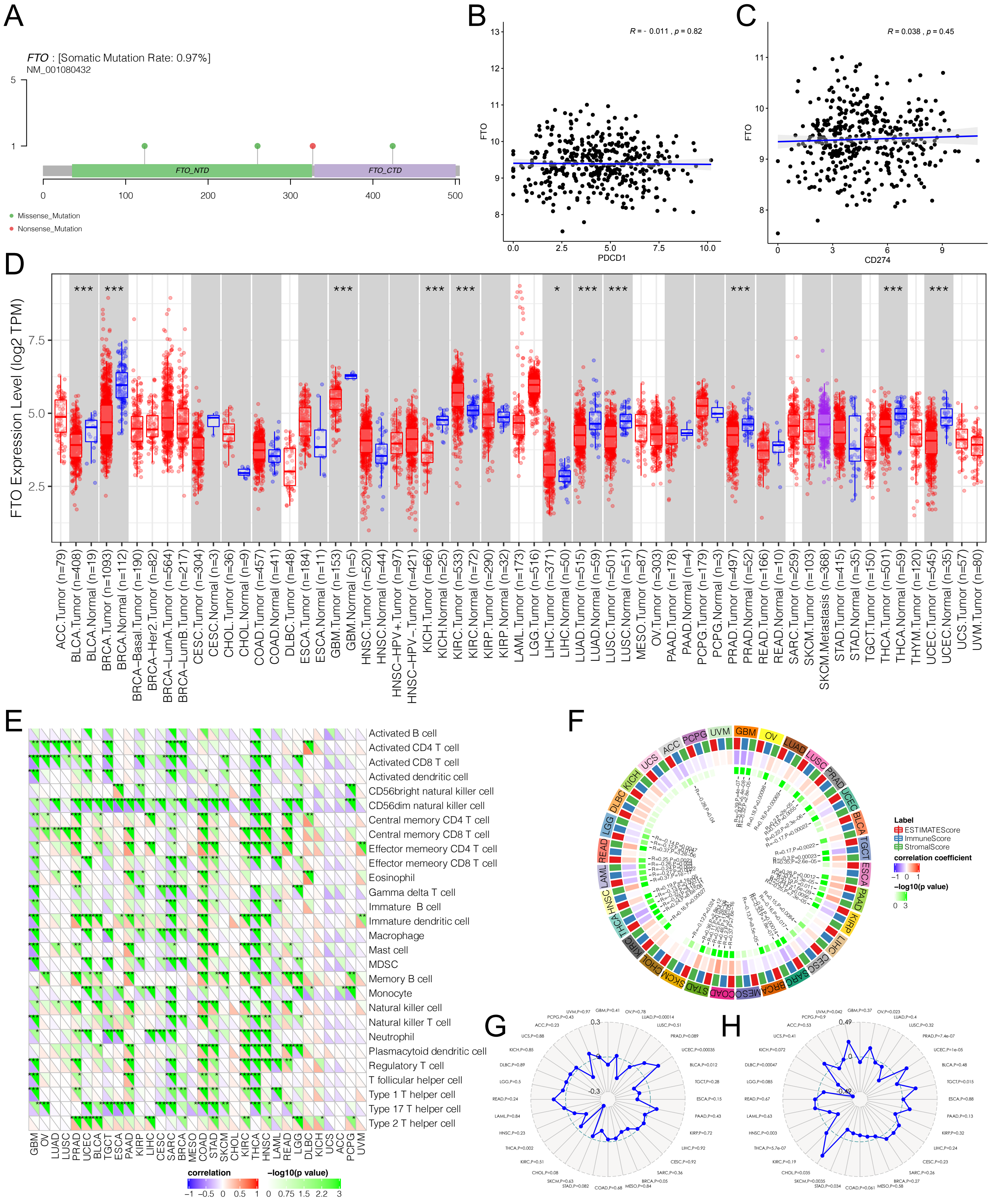
A
FTO : [Somatic Mutation Rate: 0.97%]
NM_001080432
FTO_NTD
FTO_CTD
Missense_Mutation
Nonsense_Mutation
B
R = - 0.011 , p = 0.82
FTO
PDCD1
C
R = 0.038 , p = 0.45
FTO
CD274
D
FTO Expression Level (log2 TPM)
ACC.Tumor (n=79)
BLCA.Tumor (n=408)
BLCA.Normal (n=19)
BRCA.Tumor (n=1093)
BRCA.Normal (n=112)
BRCA−Basal.Tumor (n=190)
BRCA−Her2.Tumor (n=82)
BRCA−LumA.Tumor (n=564)
BRCA−LumB.Tumor (n=217)
CESC.Tumor (n=304)
CESC.Normal (n=3)
CHOL.Tumor (n=36)
CHOL.Normal (n=9)
COAD.Tumor (n=457)
COAD.Normal (n=41)
DLBC.Tumor (n=48)
ESCA.Tumor (n=184)
ESCA.Normal (n=11)
GBM.Tumor (n=153)
GBM.Normal (n=5)
HNSC.Tumor (n=520)
HNSC.Normal (n=44)
HNSC−HPV+.Tumor (n=97)
HNSC−HPV−.Tumor (n=421)
KICH.Tumor (n=66)
KICH.Normal (n=25)
KIRC.Tumor (n=533)
KIRC.Normal (n=72)
KIRP.Tumor (n=290)
KIRP.Normal (n=32)
LAML.Tumor (n=173)
LGG.Tumor (n=516)
LIHC.Tumor (n=371)
LIHC.Normal (n=50)
LUAD.Tumor (n=515)
LUAD.Normal (n=59)
LUSC.Tumor (n=501)
LUSC.Normal (n=51)
MESO.Tumor (n=87)
OV.Tumor (n=303)
PAAD.Tumor (n=178)
PAAD.Normal (n=4)
PCPG.Tumor (n=179)
PCPG.Normal (n=3)
PRAD.Tumor (n=497)
PRAD.Normal (n=52)
READ.Tumor (n=166)
READ.Normal (n=10)
SARC.Tumor (n=259)
SKCM.Tumor (n=103)
SKCM.Metastasis (n=368)
STAD.Tumor (n=415)
STAD.Normal (n=35)
TGCT.Tumor (n=150)
THCA.Tumor (n=501)
THCA.Normal (n=59)
THYM.Tumor (n=120)
UCEC.Tumor (n=545)
UCEC.Normal (n=35)
UCS.Tumor (n=57)
UVM.Tumor (n=80)
E
Activated B cell
Activated CD4 T cell
Activated CD8 T cell
Activated dendritic cell
CD56bright natural killer cell
CD56dim natural killer cell
Central memory CD4 T cell
Central memory CD8 T cell
Effector memeory CD4 T cell
Effector memeory CD8 T cell
Eosinophil
Gamma delta T cell
Immature B cell
Immature dendritic cell
Macrophage
Mast cell
MDSC
Memory B cell
Monocyte
Natural killer cell
Natural killer T cell
Neutrophil
Plasmacytoid dendritic cell
Regulatory T cell
T follicular helper cell
Type 1 T helper cell
Type 17 T helper cell
Type 2 T helper cell
GBM OV LUAD LUSC PRAD UCEC BLCA TGCT ESCA PAAD KIRP LIHC CESC SARC BRCA MESO COAD STAD SKCM CHOL KIRC THCA HNSC LAML READ LGG DLBC KICH UCS ACC PCPG UVM
correlation
−log10(p value)
F
Label
ESTIMATEScore
ImmuneScore
StromalScore
correlation coefficient
−log10(p value)
G
H
